# Tail-specific protease (Tsp)-mediated envelope remodeling and beta-lactam tolerance in *Escherichia coli*

**DOI:** 10.1128/spectrum.00277-26

**Published:** 2026-06-26

**Authors:** Jenet Narzary, Sayed Golam Mohiuddin, Aslan Massahi, Mehmet A. Orman

**Affiliations:** 1 Department of Chemical and Biomolecular Engineering, University of Houston 684748, Houston, Texas, USA; 2 Department of Biomedical Engineering, University of Wisconsin 5228, Madison, Wisconsin, USA; National Institute of Science Education and Research, Jatni, Odisha, India

**Keywords:** bacterial persisters, Tsp protease, *Escherichia coli*, beta-lactam antibiotics, cell envelope-associated proteins

## Abstract

Bacterial persisters are transiently drug-tolerant cells that survive antibiotic treatment without genetic resistance. While certain cytosolic proteases are known to regulate persistence through toxin-antitoxin and stress pathways, the role of envelope-associated proteolysis remains poorly understood. Here, our high-throughput promoter-reporter screen identified several candidate proteases, and subsequent antibiotic treatment of knockout strains lacking these degradative genes revealed that Tsp plays a critical role in beta-lactam persistence. The deletion of *tsp* markedly reduced cell survival to multiple cell-wall-targeting antibiotics. This loss of *tsp* also compromised cell envelope integrity and caused the accumulation of periplasmic, membrane-associated, and cell-wall-related proteins, including the DD-endopeptidase MepS, as shown by quantitative proteomics. Comprehensive genetic deletion and overexpression analyses revealed that both loss and excessive activity of envelope-associated proteins, particularly MepS, compromise persistence. Moreover, cell envelope permeabilization and antibiotic sensitivity can be independent, as certain envelope perturbations increased permeability without reducing beta-lactam survival. Altogether, our findings support a model in which Tsp-mediated proteolysis helps prevent aberrant accumulation of peptidoglycan-remodeling proteins that would otherwise destabilize the envelope.

**IMPORTANCE:**

Our findings establish Tsp as an important envelope-associated protease that contributes to beta-lactam persistence by modulating peptidoglycan remodeling through proteolytic control. Loss of Tsp disrupts the balance between synthesis and degradation of cell envelope components, leading to envelope destabilization, induction of envelope-stress response, and loss of beta-lactam tolerance. By identifying multiple Tsp-regulated envelope determinants and linking envelope proteostasis to persistence, this work expands the conceptual framework of bacterial tolerance beyond cytosolic stress pathways to include periplasmic proteolytic regulation as an important layer of persistence control.

## INTRODUCTION

Persister cells are a rare subpopulation of bacteria that survive antibiotic treatment through reversible, non-genetic adaptations rather than heritable mutations (1). Unlike resistant cells, which acquire stable genetic changes that increase minimum inhibitory concentrations (MICs) and provide long-term protection, persisters survive antibiotic treatments transiently and can revert to a drug-sensitive state when the treatments are discontinued (2–4). Understanding persister physiology is, therefore, crucial, as these cells underlie relapse and treatment failure and represent a major barrier to the eradication of infectious diseases (5–8).

The tolerance of persisters arises stochastically or in response to environmental stressors, including oxidative damage, nutrient deprivation, or activation of global stress responses such as the SOS pathway and stringent response, as well as toxin–antitoxin (TA) modules that degrade or inhibit critical cellular processes (9–14). Several Type I TA systems, which consist of a small toxin protein controlled by a cis-encoded antisense RNA antitoxin, have been linked to persistence. In *E. coli*, the SOS-inducible membrane-depolarizing toxin TisB promotes fluoroquinolone tolerance by disrupting membrane potential (14). In *Staphylococcus aureus,* a chromosomally encoded tripartite type I system modulates persister formation and alters fluoroquinolone susceptibility through the regulation of the multidrug efflux pump NorA (15), further supporting a link between membrane-associated toxins and antibiotic tolerance. In addition to type I systems, type II toxins, such as HipA, MazF, RelE, MqsR, and YoeB, also promote persistence by halting translation or interfering with essential metabolic mechanisms, thus driving cells into a growth-arrest state that reduces antibiotic lethality (16–21). Importantly, the activation of these toxins is tightly regulated by ATP-dependent proteases, including Lon and ClpXP, which degrade the cognate antitoxins under stress conditions, thereby liberating the toxins to act (17, 22–24). In fact, proteases may exhibit notable complexity, acting both as activators and regulators of persistence. For instance, in our recent work, we found that the deletion of *lon* impaired persister resuscitation through a SulA-FtsZ-dependent mechanism (25), underscoring that proteolysis not only triggers cell cycle arrest via antitoxin degradation but also is critical for recovery from the persister state.

We are particularly interested in the role of proteases in bacterial persistence in this study. These enzymes are highly complex and functionally versatile, often affecting multiple cellular processes, including metabolism, envelope maintenance, and stress response regulation (26, 27). Because protease activity is closely tied to physiological state and environmental conditions, their contributions to persistence are likely context dependent and require systematic investigation. To address this, we screened the promoters of all known genes encoding degradative enzymes to identify candidates with elevated activity under our experimental conditions. This screen identified several cytosolic and periplasmic proteases with increased promoter activity. Among these, the periplasmic protease Tsp (Prc) emerged as a particularly intriguing candidate, and further analysis demonstrated its connection to envelope proteostasis, peptidoglycan remodeling, and tolerance to cell-wall-targeting antibiotics.

## RESULTS

### Promoter screening identifies Tsp as an important protease mediating ampicillin persistence

To identify degradative enzymes that may contribute to persistence or antibiotic tolerance, we examined promoter activity of selected genes encoding proteases and RNases with established macromolecule-degrading activity using an *E. coli* MG1655 promoter-GFP reporter library (28). TA modules were included not as degradative enzymes *per se*, but as persistence-associated regulatory systems functionally linked to proteolysis or RNA degradation pathways. Cultures were grown in 96-well plates and diluted into fresh LB, and GFP fluorescence was monitored over 24 h (see Materials and Methods). All reporter strains exhibited similar growth dynamics (Fig. S1). Background fluorescence from medium and empty-vector controls was subtracted, and early time points with near-background signal were not considered in downstream analyses.

**Fig 1:**
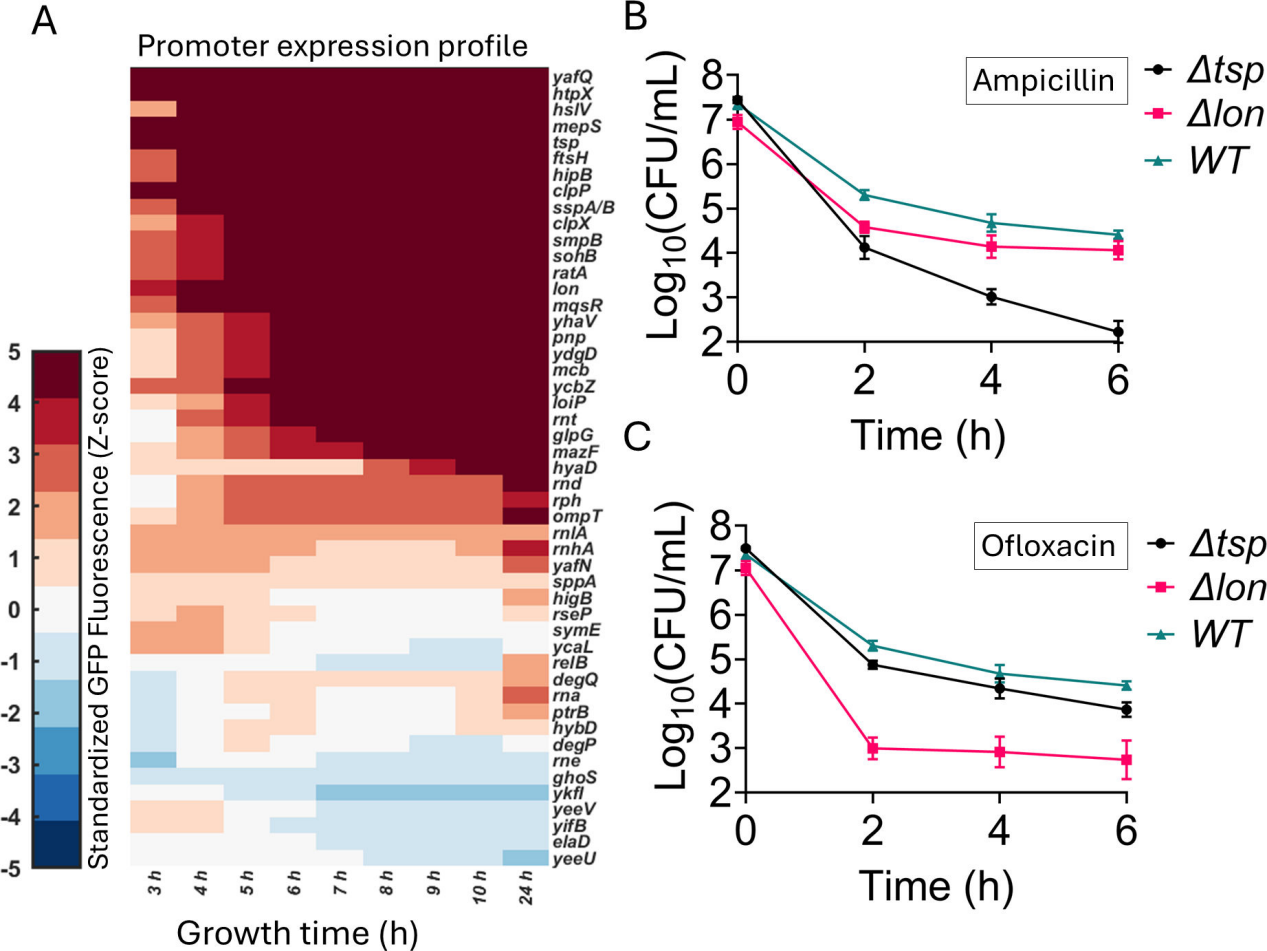
Promoter activity and persistence phenotypes of degradative enzymes. (**A**) Hierarchical clustering heatmap of promoters corresponding to genes encoding proteases and other degradative enzymes. Colors represent relative expression levels on a continuous scale from high (red) to low (blue), and promoters were clustered based on similarity in expression trajectories, revealing discrete activity classes. *n* = 1, biological replicate. (**B and C**) Persister assays of selected deletions corresponding to degradative enzyme genes. Stationary-phase cultures were diluted 100-fold into fresh LB and treated with ampicillin (200 μg/mL) (**B**) or ofloxacin (5 μg/mL) (**C**) for 6 h. Colony-forming units (CFUs) were quantified at 0, 2, 4, and 6 h by washing, serial dilution, and plating. Wild type (WT) was used as a control. Note that only the kill curves for *tsp* and *lon* knockout strains are shown in these plots; see Fig. S3 for the kill curves of all selected knockouts. *n* = 4; data represent mean ± standard deviation.

Genes selected for further analysis were prioritized based on overall cumulative promoter activity under identical experimental conditions (Fig. 1A), and the top-ranked promoters (~19 genes) were advanced to persistence assays. This distinct subset consistently exhibited higher fluorescence intensity across the measurement period relative to other candidates (Fig. 1A). Because the reporters use stable GFP, the measured signal reflects cumulative promoter output rather than instantaneous transcriptional changes; therefore, promoter activity was interpreted comparatively rather than as evidence of phase-specific induction. Small late increases observed for certain non-selected promoters reflect relative changes from low baseline levels and can visually appear slightly upregulated in the normalized heat map. However, the examination of the temporal fluorescence profiles (Fig. S2) shows that these promoters exhibit only modest signal changes and plateau over time; accordingly, they were not prioritized for further analysis.

Representative functions in the upregulated subset (see Table S1) include periplasmic/IM protein quality control (e.g., HtpX-like protease activity), AAA+ proteolysis modules (e.g., ClpP/ClpX/HslV components), peptidoglycan remodeling (e.g., the DD-endopeptidase MepS), RNA turnover (e.g., PNPase/pnp), and toxin–antitoxin elements with degradative activities (e.g., YafQ, YhaV, MqsR), as well as the tmRNA/SmpB system and the periplasmic protease Tsp (Prc) that connects envelope proteostasis to antibiotic responses (29). We performed a second screen in which the most highly induced degradative genes in *E. coli* MG1655 were deleted, and the resulting strains were subjected to persister assays (Fig. 1B and C; see Fig. S3 for the kill curves of all selected knockouts). The rationale was that if these enzymes promote persister formation, their loss should impair the fraction of surviving cells after antibiotic challenge. To test this, cultures were grown to the stationary phase and diluted into fresh LB containing 200 µg/mL ampicillin (a cell wall biosynthesis inhibitor) or 5 µg/mL ofloxacin (a DNA-damaging agent), and survival was monitored (Fig. 1B and C; Fig. S3). All knockouts displayed normal growth, confirming that differences in survival reflected persistence rather than growth defects (Fig. S4). Although most knockouts did not alter persistence, the deletion of *lon* markedly decreased ofloxacin persisters but had no effect on ampicillin persistence (Fig. 1C). In contrast, the deletion of *tsp* (*prc*) significantly reduced ampicillin persisters without altering ofloxacin tolerance (Fig. 1B and C), suggesting that Tsp influences persistence through envelope-associated processes rather than global stress or DNA repair mechanisms, which is yet to be determined. These results indicate that distinct proteases govern antibiotic-specific persistence that may be linked to cytosolic stress adaptation or cell envelope proteostasis. Other proteases and toxins identified in our screen, including the Clp complex, YafQ, and MqsR, are already known to affect persistence (20, 30–33); however, single deletions showed no clear phenotype under the conditions studied here (Fig. S3). Since the mechanisms related to these proteins, including Lon (34, 35), are well studied, our study centers on elucidating Tsp’s distinct contribution to persister physiology.

### Loss of Tsp compromises envelope integrity and reduces persistence to diverse cell wall-targeting antibiotics

**Fig 2:**
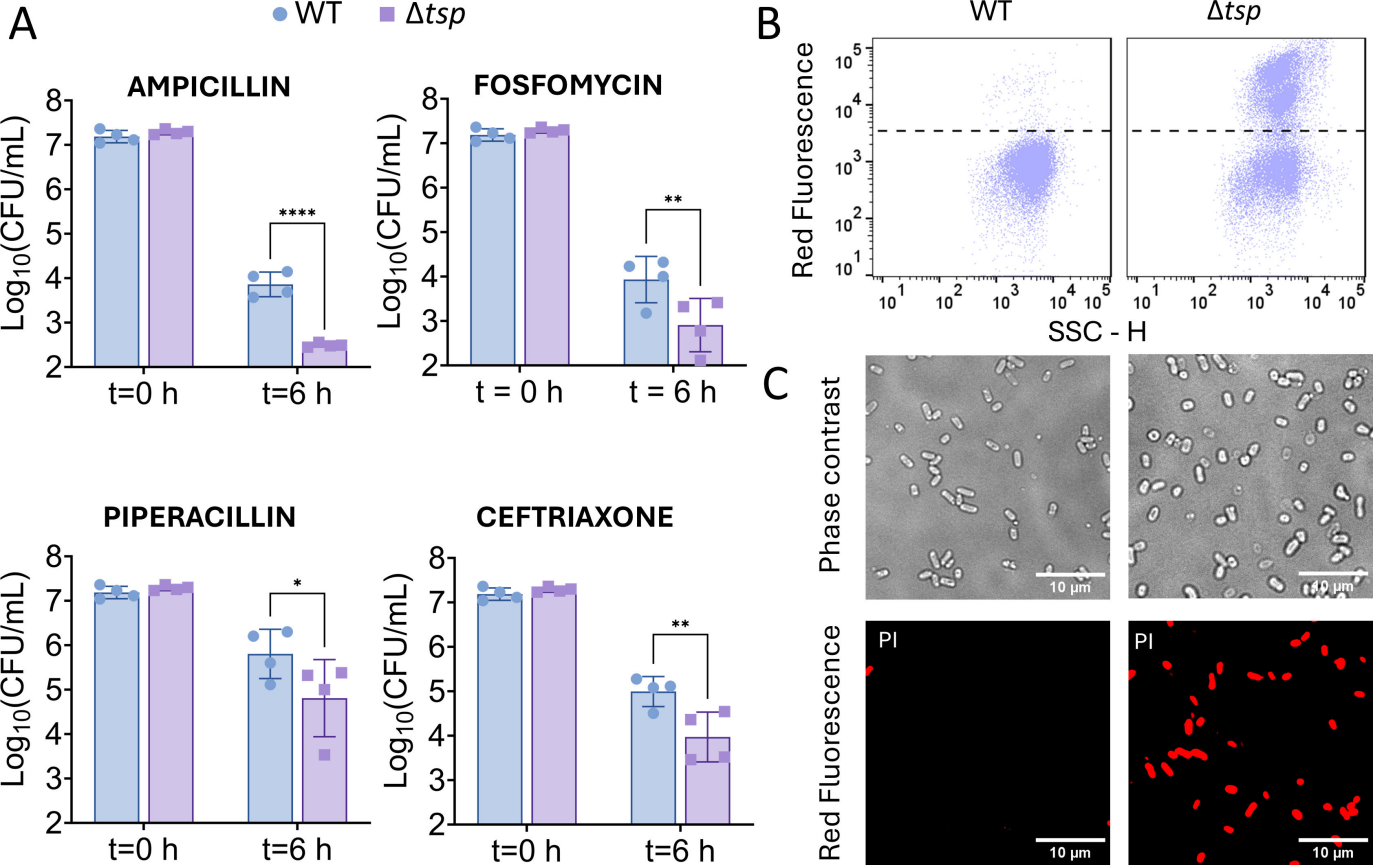
Loss of Tsp reduces persistence to cell wall-targeting antibiotics and increases envelope permeabilization. (**A**) Survival of wild-type and Δ*tsp* cultures exposed to cell wall-targeting antibiotics during late stationary phase. Stationary-phase cells were treated with ampicillin (200 μg/mL), piperacillin (200 μg/mL), fosfomycin (200 μg/mL), or ceftriaxone (5 μg/mL), and CFUs were measured before and after treatment. Statistical significance was calculated relative to wild-type (*P < 0.05, **P < 0.01, ****P < 0.0001; two-way ANOVA with Dunnett’s multiple comparisons test). *n* = 4; data represent mean ± standard deviation. (**B and C**) Envelope permeability of wild-type and Δ*tsp* cultures prior to antibiotic exposure, quantified by propidium iodide (PI) staining and analyzed by flow cytometry (**B**) and fluorescence microscopy (**C**). Representative cytometry plots and phase-contrast and red-fluorescence images are shown, with similar outcomes observed across biological replicates. Side scatter height (SSC-H) represents the peak amplitude of the side-scattered light pulse generated. *n* = 4.

Tsp is a periplasmic tail-specific protease, and because its activity might be linked to cell envelope maintenance, we examined whether Tsp affects persistence across different cell-wall targeting antibiotics, including piperacillin, fosfomycin, and ceftriaxone, which target peptidoglycan synthesis through both overlapping and distinct mechanisms. MIC measurements revealed no major difference between the *tsp* knockout (Δ*tsp*) and wild type (Table S2), indicating that Tsp deletion does not alter population-level β-lactam susceptibility. However, when cultures were challenged at concentrations well above the MIC, consistent with standard persister assays (10, 36–38), the *tsp* knockout consistently exhibited reduced persister levels across all tested β-lactams (Fig. 2A). Ampicillin and piperacillin inhibit the transpeptidase-mediated cross-linking of peptidoglycan strands; ceftriaxone targets penicillin-binding proteins (PBPs) involved in late-stage cell wall synthesis; and fosfomycin inhibits MurA, blocking the first cytoplasmic step in precursor formation (39, 40). The uniform reduction in persistence despite these mechanistic differences suggests that Tsp contributes to a general envelope-associated tolerance pathway.

Since Tsp targets periplasmic proteins, its loss might compromise cell envelope integrity. To test this, we measured cell permeability using propidium iodide (PI) staining with flow cytometry for both wild-type and the *tsp* knockout cultures grown to late stationary phase (Fig. 2B; Fig. S5 for PI-positive controls). The *tsp* knockout showed a marked increase in PI-positive cells, with approximately 50% of the population exhibiting PI uptake, whereas only a small fraction of wild-type cells was stained (Fig. 2B), and microscopy further confirmed these results (Fig. 2C). To further validate that this phenotype was not specific to a single staining approach, we performed an additional permeability assay using SYTOX Green, another membrane-impermeable nucleic acid dye. Consistent with the PI measurements, the *tsp* knockout exhibited substantially elevated SYTOX Green uptake relative to wild type (Fig. S6), supporting the conclusion that loss of *tsp* is associated with increased envelope permeability. Although this increase could, in principle, enhance drug uptake or impose additional stress leading to broader antibiotic sensitivity, we did not observe any effect of *tsp* deletion on ofloxacin persistence (Fig. 1C). This phenotype likely reflects Tsp’s specific role in maintaining envelope integrity; we, therefore, further examine how *tsp* deletion perturbs β-lactam-specific persistence mechanisms in the following sections.

To confirm that the reduced persistence and increased membrane permeabilization in the *tsp* knockout were directly caused by loss of Tsp function, we performed a complementation experiment using an isopropyl β-D-1-thiogalactopyranoside (IPTG)-inducible overexpression plasmid in which *tsp* was placed under the control of the strong T5 promoter (see Materials and Methods). IPTG was maintained throughout all growth conditions, including overnight cultures, antibiotic treatment, and solid media, to ensure constitutive induction. The complemented Δ*tsp* strain, as well as the corresponding empty-vector control, were compared with wild-type cells carrying either the empty vector or the *tsp* expression plasmid (Fig. S7). Overexpressing *tsp* in the Δ*tsp* background restored antibiotic persistence to levels comparable to the wild-type controls (Fig. S7A) and significantly reduced membrane permeabilization (Fig. S7B and C). Testing two different IPTG concentrations (0.1 and 1 mM) yielded similar restoration of antibiotic persistence and reduction in membrane permeabilization, confirming that the phenotypes observed in the knockout strain arise directly from the absence of Tsp.

### Deletion of *mepS* and *ssrA* further reduces persistence in the *tsp* knockout

Accumulated evidence suggests that Tsp functions within multiple, overlapping proteolytic pathways involving NlpI, SsrA, MepS, CsgA, and FtsI. The adaptor NlpI may recruit Tsp to specific envelope substrates (41), including MepS, a DD-endopeptidase that cleaves peptidoglycan cross-links during cell growth. The PDZ domain of Tsp mediates substrate recognition by binding nonpolar C-terminal residues such as the SsrA degradation tag (42), which marks incompletely translated or stalled proteins for degradation. Tsp also degrades other periplasmic proteins, including CsgA, the curli amyloid subunit that otherwise forms periplasmic aggregates (43), and truncates FtsI (PBP3), a transpeptidase required for septal peptidoglycan synthesis (44); however, both the truncated and untruncated versions of FtsI remain functional (45, 46).

To examine how these Tsp-associated pathways influence persistence, we deleted *nlpI*, *ssrA*, *mepS*, and *csgA* individually and in combination with *tsp*. Because NlpI and SsrA function upstream of Tsp-mediated proteolysis, we initially hypothesized that their deletion might produce persistence phenotypes similar to the *tsp* mutant if they acted within the same pathway. Conversely, because MepS and CsgA are substrates degraded by Tsp, we tested whether their removal in the Δ*tsp* background could restore persistence. The deletion of *ftsI* was not examined due to its essentiality, but its mechanism was explored in subsequent sections.

**Fig 3:**
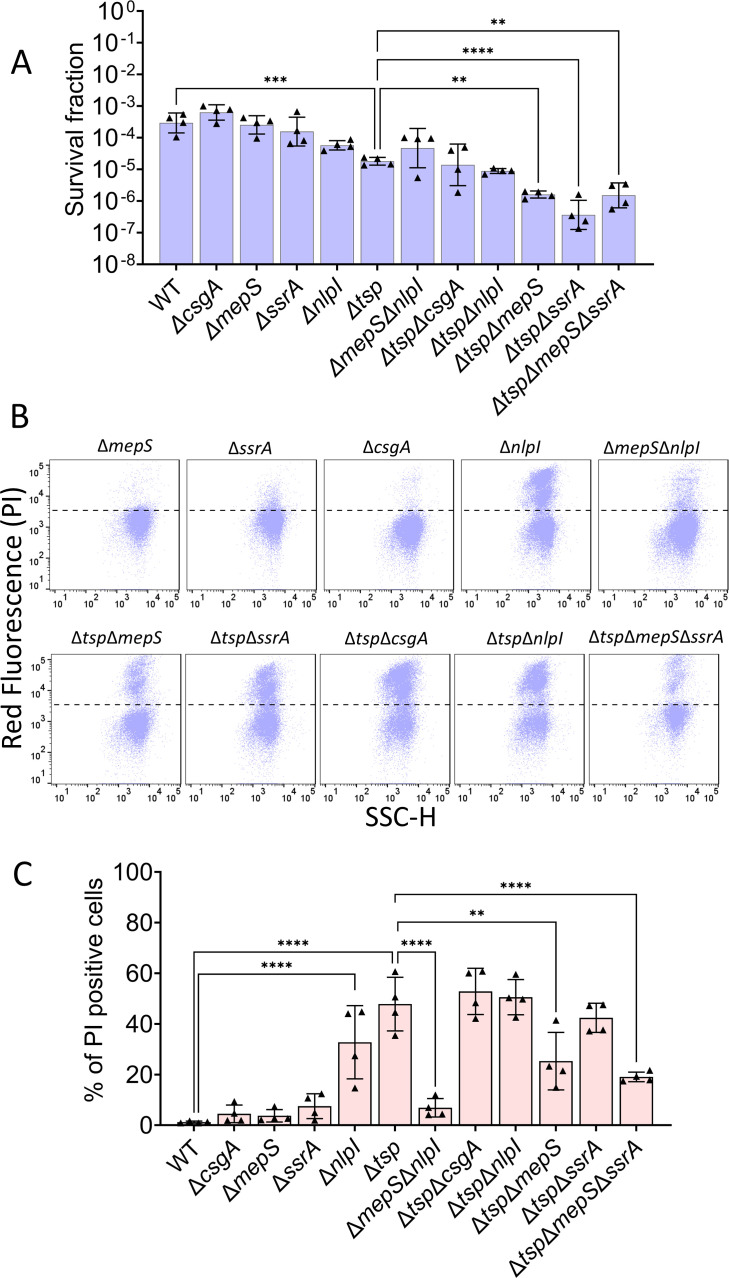
Effects of combined *tsp* and envelope-remodeling gene deletions on antibiotic survival and envelope integrity. (**A**) Survival of single, double, and triple knockout strains following ampicillin treatment. Stationary-phase cultures were exposed to 200 μg/mL ampicillin. Survival was calculated as the CFU ratio before and 6 h after treatment. Full kill curves are shown in Fig. S8. (**B and C**) Envelope permeability of stationary-phase cultures assessed by PI staining and analyzed by flow cytometry. (**B**) Representative flow cytometry plots are shown. (**C**) Quantification of PI-positive cells. Statistical significance was calculated between wild-type and single knockouts and between Δ*tsp* and combinatorial knockouts (**P < 0.01, ***P < 0.001, ****P < 0.0001; one-way ANOVA with Dunnett’s multiple comparisons test). *n* = 4; data represent mean ± standard deviation.

However, the results differed from our initial expectation of epistasis or rescue. The deletion of *nlpI* caused only a mild, non-significant reduction in ampicillin persistence, and the Δ*tspΔnlpI* mutant did not further reduce survival relative to Δ*tsp* (Fig. 3A; Fig. S8 for kill curves). Similarly, the deletion of *csgA*, alone or in combination with *tsp*, had no effect on persistence (Fig. 3A). In contrast, although single deletions of *mepS* or *ssrA* did not affect persistence, the Δ*tsp*Δ*mepS*, Δ*tsp*Δ*ssrA*, and Δ*tsp*Δ*mepS*Δ*ssrA* mutants exhibited significantly lower persister levels than Δ*tsp* alone (Fig. 3A), indicating enhanced killing rather than phenotypic rescue.

We next assessed cell envelope integrity in the single and combinatorial knockout strains using PI staining (Fig. 3B and C; Fig. S9 for PI-positive controls; Fig. S10 for microscopy images). All double and triple mutants containing the *tsp* deletion showed elevated PI uptake, confirming that loss of Tsp increases envelope permeabilization (Fig. 3B and C). In contrast, single deletions of *mepS*, *ssrA*, or *csgA* did not affect permeability. The deletion of *nlpI* alone significantly increased permeability without altering antibiotic survival, but the Δ*tspΔnlpI* mutant did not show further permeabilization beyond Δ*tsp* (Fig. 3B and C). In contrast, the deletion of *mepS* reduced PI uptake in the Δ*nlpI* background, suggesting a rescuing effect of *mepS* deletion (Fig. 3C). Similarly, the deletion of *mepS* in mutants containing the *tsp* deletion had a rescuing effect; however, envelope integrity was only partially restored (Fig. 3C).

To determine whether this phenotype extended beyond ampicillin, we tested the double and triple knockouts against additional cell wall-targeting antibiotics (piperacillin, fosfomycin, and ceftriaxone). Mutants that showed reduced persistence to ampicillin exhibited similarly decreased survival with these antibiotics (Fig. S11), indicating that the enhanced killing reflects broader defects in cell envelope-associated tolerance. Altogether, these outcomes differ from the expected epistasis or rescue, suggesting that Tsp-linked persistence does not operate through a single linear pathway (e.g., NlpI → Tsp → MepS or Tsp → SsrA-tagged proteins), but through partially parallel processes affecting the envelope during β-lactam stress. Consistent with this, the deletion of *mepS* did not fully rescue permeability defects in mutants containing the *tsp* deletion, indicating that additional Tsp-dependent pathways contribute to envelope homeostasis.

### Comparative proteomics identifies envelope and peptidoglycan remodeling defects in the *tsp* knockout

**Fig 4:**
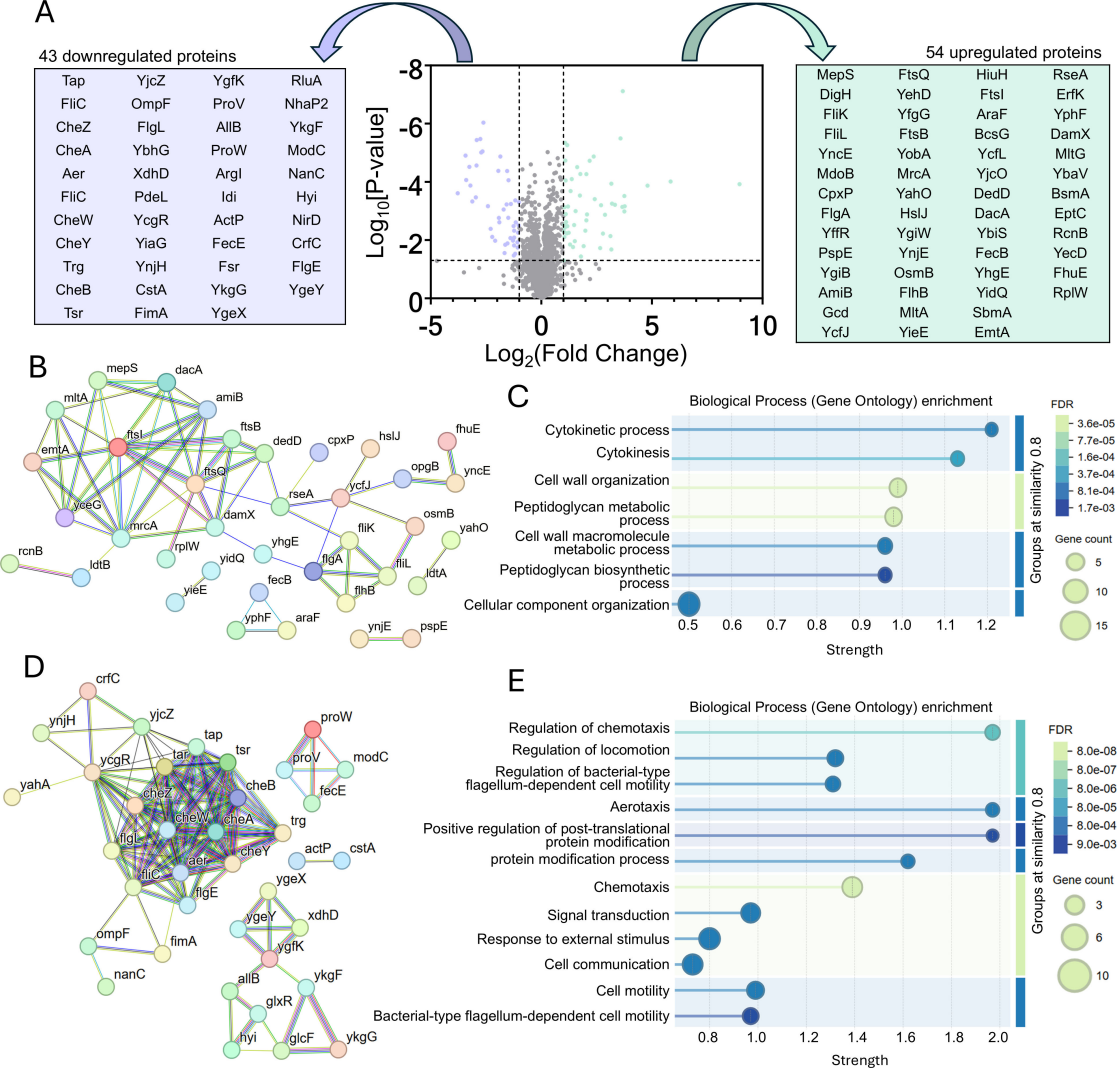
Proteomic profiling reveals remodeling of envelope- and division-associated pathways in Δ*tsp* during late stationary phase. (**A**) Volcano plot showing differentially expressed proteins in Δ*tsp* compared with wild type during late stationary phase. Upregulated proteins are shown in green and downregulated proteins in purple. Statistical significance was determined using two-tailed *t*-tests (see reference 48 for details), with thresholds set at fold-change ≥ 2 or ≤ −2 and adjusted *P* value < 0.05. *n* = 3. (**B**) STRING network of proteins upregulated in Δ*tsp*. Nodes represent proteins, and edges indicate associations visualized in evidence mode. Proteins clusters correspond to envelope remodeling, peptidoglycan hydrolases, divisome components, and stress-associated pathways. *n* = 3. (**C**) Functional pathway enrichment analysis of upregulated proteins. Enrichment reflects observed vs expected associations (STRING enrichment score), with statistical significance determined using FDR-corrected *P* values (Benjamini–Hochberg). *n* = 3. (**D**) STRING network of proteins downregulated in Δ*tsp*, shown as in panel B. Protein clusters include motility and chemotaxis pathways, transport systems, and signaling components. *n* = 3. (**E**) Functional pathway enrichment analysis of downregulated proteins. Enrichment reflects observed vs expected associations (STRING enrichment score), with statistical significance determined using FDR-corrected *P* values (Benjamini–Hochberg). *n* = 3.

To determine these additional molecular players, we performed a comparative proteomic analysis of wild-type and Δ*tsp* cells collected from stationary-phase cultures, consistent with persister assay conditions. Our goal was to identify global changes in protein abundance that could reveal downstream pathways affected by the loss of Tsp-mediated proteolysis. Whole-cell lysates were analyzed by quantitative mass spectrometry, and relative protein abundances were compared between strains. Differentially expressed proteins were visualized using volcano plots to highlight statistically significant fold changes, with thresholds set at an adjusted *P* value < 0.05 and a minimum twofold difference (Fig. 4A). Functional enrichment of the upregulated and downregulated proteins was then evaluated using the STRING database (47) (see Materials and Methods). Enrichment scores were calculated as the log10 ratio of observed vs expected proteins per category, and significance was determined using false discovery rate-corrected *P* values (47). Proteomic profiling revealed that loss of Tsp triggered broad remodeling of the *E. coli* proteome during late stationary phase (Fig. 4A). Among the significantly altered proteins, those involved in envelope homeostasis, peptidoglycan metabolism, and periplasmic proteostasis were strongly upregulated (Fig. 4B and C), consistent with the activation of compensatory stress pathways in response to defective proteolytic processing. In contrast, proteins associated with chemotaxis, motility, post-translational modification, and cell communication or signal transduction were markedly downregulated (Fig. 4D and E), indicating a global shift from active environmental adaptation to a stress-maintenance state. Network enrichment analysis (Fig. 4B through E) confirmed these trends, showing enrichment for categories related to cell envelope organization and proteolysis among upregulated proteins and depletion of motility and signaling functions among downregulated ones.

**Fig 5:**
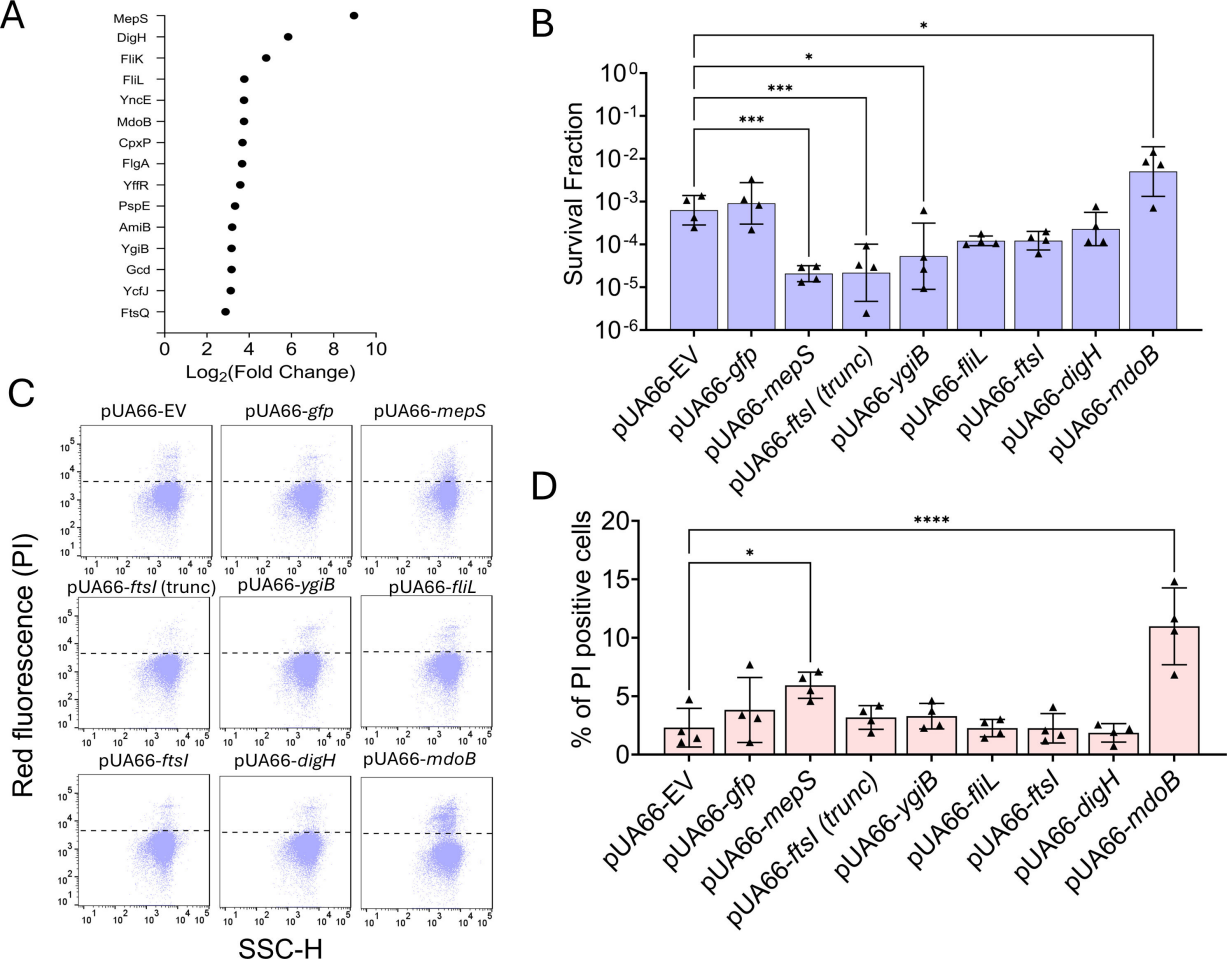
Overexpression of selected envelope-associated proteins affects persistence and envelope permeability. (**A**) Fold-change values of selected proteins upregulated in Δ*tsp* identified from the proteomics data set. Only the most upregulated proteins are shown; see Table S3 for the full list. *n* = 3. (**B**) Survival of stationary-phase cultures overexpressing selected envelope-associated proteins following ampicillin treatment (200 μg/mL). Survival was calculated as the CFU ratio before and 6 h after treatment. Full kill curves are shown in Fig. S13. Empty-vector and GFP-expressing plasmids were used as controls. (**C**) Representative flow cytometry plots showing PI uptake in overexpression strains prior to antibiotic treatment. *n* = 4. (**D**) Quantification of PI-positive fractions corresponding to panel C. Empty-vector and GFP-expressing plasmids were included as controls. Statistical significance was calculated relative to the empty-vector control (*P < 0.05, ***P < 0.001, ****P < 0.0001; one-way ANOVA with Dunnett’s multiple comparisons test). *n* = 4; data represent mean ± standard deviation.

Because Tsp is a periplasmic protease responsible for degrading envelope-associated proteins, its loss is expected to reduce turnover and thereby increase the accumulation of its direct or indirect substrates. We, therefore, focused our analysis on proteins upregulated in Δ*tsp*, reasoning that these may represent Tsp targets or compensatory factors involved in maintaining envelope homeostasis (Fig. 5A; Table S3). The most strikingly elevated protein was MepS, a DD-endopeptidase that cleaves peptide cross-links in peptidoglycan to allow cell wall expansion (log₂ fold change >8) (Fig. 5A). DigH, a divisome-associated hydrolase involved in septal peptidoglycan cleavage (49), was also highly upregulated (log₂ fold change ≈ 6), together with AmiB, another septal amidase essential for daughter-cell separation (50) (≈3-fold) (Fig. 5A). These changes collectively indicate dysregulation of cell wall remodeling enzymes. In addition to these peptidoglycan-associated proteins, several flagellar assembly components, including FliK (a hook-length control protein [51]) and FliL (a basal-body-associated protein that stabilizes flagellar rotation [52]), were also elevated (~3- to 5-fold) (Fig. 5A). The membrane-bound protein MdoB, a phosphoglycerol transferase involved in osmoregulated periplasmic glucan modification (53), and YgiB, an outer membrane protein associated with biofilm formation and envelope stress responses (54), were also significantly upregulated (~3- to 4-fold) (Fig. 5A). Other enriched proteins included cell division factors FtsQ, FtsB, and FtsI (PBP3), along with murein transglycosylases (MltA, MltE, MltG) and L, D-transpeptidases (LdtA, LdtB), reflecting a broader reorganization of the peptidoglycan synthesis and turnover network (Fig. 5A; Table S3). Together, these findings indicate that Tsp deletion redundantly affects many envelope-associated factors, providing a plausible explanation for the increased membrane permeability, altered antibiotic survival, and accumulation of envelope-associated proteins observed in the Δ*tsp* strain.

### Overexpression of envelope-associated proteins differentially impacts persistence and envelope integrity

To analyze the contribution of key upregulated proteins to persistence and permeabilization, we generated a series of overexpression plasmids in which each target gene was placed under the IPTG-inducible T5 promoter, constructed and maintained under the same induction conditions used for the *tsp* complementation experiment. These plasmids were expressed in wild-type *E. coli*, and IPTG was present throughout all growth stages to ensure constitutive induction. Our rationale is that if increased levels of these proteins drive the phenotypes observed in the Δ*tsp* mutant, then overexpressing them in a wild-type background should recapitulate similar persistence and permeabilization phenotypes. Based on the analysis of the proteomics data, we selected the proteins with the highest fold-change compared to wild-type, MepS and DigH (Fig. 5A), both involved in peptidoglycan cleavage and remodeling, as primary candidates. In addition, several periplasmic or membrane-associated proteins were also selected (YgiB, MdoB, FliL) to test whether enhanced expression of general envelope components also affects persistence and envelope integrity. We also included both the full-length and truncated forms of FtsI (PBP3) (see Materials and Methods). Truncation of FtsI results from Tsp-mediated C-terminal processing, which has been shown to occur without disrupting FtsI’s essential transpeptidase function (46, 55); comparing both versions allowed us to determine whether this processing event contributes to Tsp-dependent persistence mechanisms. Each construct was individually expressed, and envelope permeabilization was quantified in the late stationary phase prior to antibiotic exposure, followed by ampicillin persister assays under the same conditions. Although we did not measure expression levels or functional activity for each protein, the same T5 promoter and plasmid backbone successfully restored persistence and membrane integrity in the *tsp* complementation experiment (Fig. S7) and also expressed functional GFP under identical induction conditions (Fig. S12), confirming that this system can reliably drive protein production.

Overexpression analysis revealed that only a subset of the tested proteins significantly altered persistence or envelope permeability. Specifically, MepS, truncated FtsI, and YgiB overexpression each resulted in approximately a 10-fold reduction in persister levels compared with the empty vector or GFP-expressing controls (Fig. 5B; Fig. S13 for the corresponding kill curves). In contrast, the overexpression of FliL, full-length FtsI, or DigH did not produce significant changes in survival, and cells overexpressing MdoB displayed a slight increase in tolerance relative to controls (Fig. 5B; Fig. S13). These findings indicate that both overexpression and loss of specific envelope enzymes can disrupt persistence through distinct mechanisms. Interestingly, while the overexpression of *mepS* reduced persistence, *mepS* deletion in the Δ*tsp* background also decreased persistence rather than rescuing it (Fig. 5B). This reveals a complex effect in which both excessive and insufficient MepS activity may impair tolerance, suggesting that tight regulation of MepS by Tsp might be critical for cell survival under stress. Also, truncated FtsI overexpression reduced persistence, whereas full-length FtsI overexpression did not, even though Δ*tsp* accumulates only the full-length form. This indicates that the presence of the truncated variant itself, rather than the lack of truncation in Δ*tsp*, perturbs β-lactam persistence.

Analysis of envelope permeability showed that MdoB overexpression significantly increased PI uptake, further supporting the involvement of additional molecular players in envelope regulation, while the overexpression of the other proteins resulted in no significant or only minor increases in permeability (Fig. 5C and D; Fig. S14 for PI-positive controls). Despite this increase in permeability, MdoB overexpression also slightly increased tolerance, demonstrating that envelope permeabilization alone does not necessarily enhance antibiotic sensitivity. This notion is also supported by our results in Fig. 3, in which *nlpI* deletion causes a drastic increase in envelope permeabilization without a significant change in persistence. Also, overexpressing *mepS* from the constructed plasmid in the wild-type background slightly increased the permeabilization (Fig. 5C and D). Altogether, these perturbations clearly show that changes in envelope protein abundance can differentially affect cellular permeability or persistence. Our results also point to functional redundancy, since no single overexpressed protein or gene deletion recapitulated the full *tsp* phenotype, which is expected given that Tsp seems to influence numerous envelope-associated pathways.

## DISCUSSION

The promoter-based screen used in this study relied on GFP reporters, which provide an integrated measure of promoter activity over time. Because GFP is relatively stable, these measurements do not necessarily reflect instantaneous transcriptional changes. We, therefore, used the promoter screen as an initial prioritization strategy, which led to the identification of a previously unrecognized role for the periplasmic protease Tsp in antibiotic persistence. Our findings indicate that loss of Tsp selectively impairs survival to multiple β-lactam antibiotics while having little effect on ofloxacin persistence, suggesting that proteolytic regulation contributes to antibiotic-specific tolerance pathways. Antibiotic treatment itself likely plays a major role in selecting or enriching tolerant subpopulations. We also observed altered membrane permeability in Δ*tsp*, which may contribute to the persistence phenotype by changing intracellular or periplasmic antibiotic exposure. However, permeability alone does not fully explain the observed tolerance defects. For example, some perturbations (e.g., Δ*nlpI* and MdoB overexpression) increased PI uptake without reducing β-lactam persistence, indicating that Tsp-dependent envelope homeostasis likely influences tolerance through more specific physiological mechanisms beyond generalized membrane permeability.

The proteomic analysis revealed a broad accumulation of envelope and peptidoglycan-modifying enzymes in Δ*tsp*. Because many of these proteins act in interconnected pathways, dissecting their combined contributions is experimentally challenging; simultaneous overexpression of multiple factors or precisely tuned expression levels would likely be required to capture their collective effects. Among these, however, MepS emerged as the most critical determinant. Its abundance increased dramatically in Δ*tsp*, and this strong enrichment is consistent with previous reports that Tsp degrades MepS in an NlpI-dependent manner (56–58); however, our results indicate that NlpI alone is not sufficient to account for the observed persistence mechanism. Both genetic and overexpression analyses demonstrated that perturbing MepS levels strongly influences persistence. Specifically, the deletion of *mepS* in the Δ*tsp* background further decreased survival, while MepS overexpression in wild-type cells similarly reduced persisters. These observations indicate that dysregulated MepS levels can disturb envelope homeostasis and that Tsp-mediated proteolysis acts to limit such disruptions under stress conditions associated with persistence.

Beyond MepS, our overexpression analyses suggest that additional Tsp-regulated proteins, including truncated FtsI and YgiB, may contribute to persistence through complementary effects on envelope remodeling and cell division. FtsI is the primary divisome-associated transpeptidase responsible for septal peptidoglycan synthesis and is a direct target of β-lactam antibiotics (59–61). Altered abundance or truncation of FtsI may, therefore, influence divisome function, septal remodeling, and antibiotic susceptibility. Because elevated MepS activity can interfere with septal peptidoglycan biogenesis by altering crosslink cleavage and divisome coordination (62), simultaneous perturbation of MepS and FtsI may create imbalances between peptidoglycan cleavage and synthesis during cell division. In parallel, YgiB has been associated with envelope-associated fitness and biofilm formation (54), while the deletion of ygiB exacerbates growth defects in strains compromised for TolC-dependent envelope homeostasis (63), suggesting a possible role in maintaining envelope stability under stress conditions.

Previous studies have demonstrated that Tsp plays a critical role in maintaining envelope integrity and bacterial survival under host-associated stresses. Wang et al. (64) showed that a *tsp* mutant of the pathogenic *E. coli* K1 strain RS218 was highly susceptible to complement-mediated serum killing, resulting in reduced bacteremia in mice (64). This phenotype was attributed to hyperactivation of the classical complement pathway and excessive membrane attack complex deposition, consistent with compromised envelope stability. More recently, Huang et al. (65) demonstrated that Tsp is essential for full uropathogenic *E. coli* virulence, where *tsp* deletion impaired urinary tract colonization by destabilizing the envelope and triggering sigmaE (RpoE)- and RcsCDB-dependent stress responses that suppress flagellar gene expression and motility (65). Together, these studies established that Tsp contributes broadly to envelope homeostasis and stress adaptation, linking its proteolytic activity to bacterial fitness and virulence. Our findings further support and extend these observations, showing that Tsp-mediated envelope proteostasis is critical not only for pathogenesis but also for antibiotic persistence.

Proteomic analysis of Δ*tsp* revealed broad upregulation of proteins associated with peptidoglycan remodeling, envelope maintenance, and cell division (Fig. 4). Among the most enriched were the DD-endopeptidase MepS, septal hydrolases such as DigH and AmiB, and division or transpeptidase factors including FtsI, FtsQ, and FtsB, all of which participate in dynamic restructuring of the cell wall during growth and stress adaptation (49, 66–69). Several murein transglycosylases (MltA, MltE, MltG) and L,D-transpeptidases (LdtA, LdtB) were also elevated, consistent with increased turnover and cross-link rearrangement of the peptidoglycan network (70–73). Together, these changes indicate that the absence of Tsp-mediated proteolysis leads to unbalanced activation of envelope biogenesis and degradation machineries, forcing the cell into a hyper-remodeling state. Mechanistically, such dysregulation increases the structural burden on the envelope, potentially weakening its capacity to withstand β-lactam-induced inhibition of peptidoglycan synthesis.

Although our proteomics data revealed that FliK and FliL were significantly upregulated in Δ*tsp*, this likely reflects a secondary compensatory response to envelope perturbation rather than the activation of the motility program. Loss of Tsp disrupts peptidoglycan remodeling, which can indirectly affect flagellar anchoring and basal-body stability, leading to altered expression of specific structural components such as FliK and FliL. In contrast, a broad set of motility and chemotaxis proteins, including FliC, FlgE, FlgL, CheA, CheB, CheW, CheY, CheZ, Tar, Tsr, and Trg, were significantly downregulated in Δ*tsp*, along with several genes linked to transport and energy metabolism (OmpF, ActP, FecE, ModC, ProV, ProW, and CstA) (Fig. 4). This global repression pattern suggests that loss of Tsp triggers an envelope-stress response that reprograms cellular physiology away from energy-intensive processes such as flagellar synthesis and chemotactic signaling. The accumulation of misfolded or unprocessed periplasmic proteins in the absence of Tsp may be linked to the Cpx and Rcs two-component stress systems, which are known to suppress *flhDC* and *fliA* regulons governing flagellar and motility gene expression (65, 74, 75). These pathways simultaneously promote the transcription of periplasmic folding and degradation factors such as *degP* and *dsbA* to restore envelope homeostasis (76). Also, the observed downregulation of chemotaxis and motility genes may not be a direct effect of Tsp loss on transcriptional machinery, but an indirect regulatory consequence of envelope stress and sigma-factor competition caused by periplasmic proteostasis imbalance (65). This adaptive reallocation of resources away from motility and signaling toward stress mitigation likely contributes to both the reduced persistence and envelope integrity defects observed in the *tsp* knockout.

### Future directions

Although the current study centers on Tsp-mediated persistence and envelope remodeling, our findings open several promising avenues for future investigation. While it is not the scope of the current study, one important direction is to determine which of the upregulated proteins identified in the proteomics analysis are direct substrates of Tsp. Beyond established Tsp-associated factors such as MepS, several less-characterized proteins highlighted in Fig. 5A, including DigH, YgiB, PspE, YncE, and others, represent promising candidates for future investigation. These proteins could be individually overexpressed and purified, followed by *in vitro* degradation assays using purified Tsp to determine whether they represent previously unrecognized direct substrates or indirect downstream effects of Tsp-dependent envelope regulation.

Another important question is how disruption of envelope proteostasis influences β-lactam tolerance. Disruption of envelope proteostasis may sensitize stationary-phase cells to β-lactams by perturbing the balance of peptidoglycan remodeling and envelope integrity during antibiotic challenge. For example, accumulation of peptidoglycan hydrolases and remodeling enzymes (e.g., MepS, DigH, AmiB, and lytic transglycosylases) could increase basal cell wall turnover, rendering cells more vulnerable when transpeptidation is inhibited. In addition, periplasmic proteostasis imbalance may activate envelope stress responses or disrupt proper assembly of envelope complexes such as the divisome or PBP machinery. Finally, alterations in outer-membrane permeability or transport processes could change local antibiotic exposure at PBPs without necessarily correlating directly with PI staining. Future studies that directly measure β-lactam accumulation, PBP target engagement, and envelope stress activation will help clarify how envelope proteostasis influences antibiotic tolerance.

## MATERIALS AND METHODS

### Bacterial strains and plasmids

Unless stated otherwise, all the experiments were conducted using *Escherichia coli* K-12 MG1655 and its knockout strains (see Table S4). The knockout strains were generated using the Datsenko and Wanner method (77). Single-gene knockout strains (Δ*tsp*, Δ*mepS*, Δ*nlpI*, Δ*ssrA*, Δ*csgA*, etc.) were constructed by replacing the target gene with a kanamycin resistance cassette using the temperature-sensitive pKD46 plasmid, which expresses the λ-Red recombination system (77). To generate double and triple knockout strains (Δ*mepS*Δ*nlpI*, Δ*tsp*Δ*csgA*, Δ*tsp*Δ*mepS*, Δ*tsp*Δ*ssrA*, Δ*tsp*Δ*nlpI,* Δ*tsp*Δ*ssrA*Δ*mepS*), the kanamycin cassette in the single-gene mutant was excised to create a scarless mutation using the temperature-sensitive pCP20 plasmid expressing Flp recombinase (77). Successful cassette removal was confirmed by PCR. The second deletion was then introduced using the same recombineering strategy, and this process was repeated to construct the triple mutant. All primers used for recombination and PCR verification are listed in Table S5. The *E. coli* MG1655 promoter reporter strain library (28), formatted in a 96-well plate for screening, was obtained from Horizon Discovery (Lafayette, CO, USA).

To construct plasmids overexpression, we used the previously described low-copy pUA66 system, in which the gene of interest was placed under an IPTG-inducible synthetic T5 promoter, supported by *lacI^q^* for tight repression and a kanamycin resistance marker for selection (37). This plasmid was generously gifted by Dr. Mark P. Brynildsen (Princeton University). To construct the overexpression plasmids (pUA66*-mepS*, pUA66-*ygiB*, pUA66*-fliL*, pUA66*-ftsI* etc.), the traditional restriction ligation cloning was performed. Briefly, the plasmid backbone and the gene of interest were digested with appropriate restriction enzymes to generate compatible sticky ends. The digested fragments were then ligated using the NEB ligation kit, and the resulting constructions were introduced into the target *E. coli* strain via chemical transformation. For the construction of the truncated *ftsI* overexpression plasmid (pUA66*-ftsI* truncated), the last 10 amino acids of FtsI were deleted based on a previous study showing that this C-terminal segment is removed by Tsp (55), and the resulting truncated coding sequence was cloned using EcoRI and BamHI restriction sites. A comprehensive list of cloning primers, along with the steps used for overexpression plasmid construction, is provided in Tables S5 and S6. All generated plasmids were fully sequenced by an external service (Plasmidsaurus, Inc., Louisville, KY, USA) to ensure their accuracy.

### Chemicals, media, and growth conditions

Unless otherwise specified, all chemicals were obtained from Fisher Scientific (Atlanta, GA) or VWR International (Pittsburgh, PA). Propidium iodide (PI) staining kits were purchased from Thermo Fisher Scientific (Waltham, MA). Standard Luria Bertani (LB) broth was prepared by dissolving 5 g yeast extract, 10 g tryptone, and 10 g NaCl in 1 L deionized (DI) water. LB agar plates were prepared by dissolving 40 g premixed LB agar powder in 1 L DI water. Both LB broth and LB agar were sterilized by autoclaving at 121°C and 103.4 kPa. For persister assays, ampicillin (200 µg/mL), fosfomycin (200 µg/mL), piperacillin (200 µg/mL), and ceftriaxone (5 µg/mL) were used at concentrations substantially higher than the minimum inhibitory concentrations (MICs), consistent with previous studies (78–80). Kanamycin (50 µg/mL) was used to maintain plasmids, and isopropyl β-D-1-thiogalactopyranoside (IPTG; 1 mM) was used to induce protein expression. Cells were washed with sterile 1 × phosphate-buffered saline (PBS) to reduce extracellular antibiotic concentrations below the MIC. For PI staining, sterile 0.85% NaCl solution was used. Antibiotics and IPTG stocks were prepared in DI water and sterilized by filtration through 0.2 µm PES syringe filters. Overnight cultures were initiated from frozen glycerol stocks (25% glycerol, stored at −80°C) by transferring a small amount of cells with a sterile pipette tip into 2 mL LB medium in 14-mL round-bottom culture tubes. Cultures were incubated for 24 h at 37°C with shaking at 250 rpm. Overnight cultures were then diluted 1:1,000 into 2 mL fresh medium in 14-mL tubes and for 24 h. We designated this culture as the main culture, which was subsequently used for assays at the late stationary phase (*t* = 24 h). We did not use overnight cultures directly for assays because their initial cell numbers are difficult to control and cells undergo resuscitation from freezing, both of which introduce substantial variability; using standardized main cultures avoids this variability and ensures consistent starting populations.

### Screening the *E. coli* promoter library

Overnight precultures were initiated by using sterile multichannel pipette tips to transfer cells from the promoter-library plates into fresh 96-well plates containing 250 µL LB medium per well. We only selected the promoters of proteins known to be proteases or degradative enzymes. Culture plates were sealed with a sterile, oxygen-permeable membrane (Breathe-Easier, Cat# BERM-2000; VWR International) and incubated at 37°C for 24 h while shaking at 250 rpm. The resulting cultures were then diluted 1:50 into fresh LB medium in a new 96-well plate, resealed with the same membrane type, and incubated under identical conditions. The optical density (OD_600_) and green fluorescent protein (GFP) expression were measured using Varioskan LUX Multimode Microplate Reader (Thermo Fisher Scientific, Waltham, MA, USA) at designated time points (*t* = 0, 1, 2, 3, 4, 5, 6, 7, 8, 9, 10, and 24 h). Plate reader data were acquired with SkanIt Software version 5.0.

Time-dependent GFP expression profiles for individual promoters were normalized by subtracting background fluorescence values obtained from *E. coli* MG1655 WT cells carrying the empty vector control (pUA66-empty). Following normalization, the processed data were subjected to unsupervised hierarchical clustering using the clustergram function in MATLAB (MathWorks, Natick, MA, USA) to visualize global expression patterns across the promoter library. We excluded data from the early time points (*t* = 0, 1, and 2 h) since the GFP fluorescence at these intervals primarily reflected background noise and false-positive signals from the LB medium, which could confound downstream analyses.

The clustergram algorithm generates a combined heatmap and dendrogram representation that reveals hierarchical relationships among promoters (rows) and experimental conditions or time points (columns). Prior to clustering, fluorescence data were standardized along rows or columns to achieve a mean of zero and a standard deviation of one, facilitating direct comparison across data sets. Pairwise distances were computed using the Euclidean distance metric, and unsupervised hierarchical clustering was performed with the average linkage method in MATLAB. The resulting heatmap used a diverging red, blue colormap, where red indicates higher relative GFP expression, blue indicates lower expression, and white represents mean values. The display range parameter (set to 5 by default) controlled the intensity scaling of standardized values. Row and column labels represented promoter identities and experimental conditions, respectively. Distinct promoter clusters identified from the dendrogram were subsequently exported for downstream analyses.

### Cell growth and MIC assays

Overnight cultures of indicated strains were diluted 1:1,000 into fresh LB medium (2 mL per tube) and returned to the shaker under the same incubation conditions (37°C, 250 rpm). Cell growth was monitored by measuring OD_600_ using a microplate reader at the indicated time points.

Minimum Inhibitory Concentrations (MICs) were determined using Liofilchem gradient diffusion strips, and values correspond to the lowest concentration that completely inhibited visible growth after 16–24 h of incubation. Only strains that exhibited reduced persistence phenotypes were included in this analysis.

### Persister assays

Overnight cultures of indicated strains were diluted 1:1,000-fold into 2 mL LB broth in 14-mL culture tubes and incubated for 24 h at 37°C with shaking (250 rpm). Stationary-phase cultures (*t* = 24 h) were then diluted 1:100 into 2 mL fresh LB medium in 14-mL tubes and treated with the following antibiotics for 6 h: ampicillin (200 µg/mL), fosfomycin (200 µg/mL), piperacillin (200 µg/mL), and ceftriaxone (5 µg/mL). At the indicated time point, 100 µL of antibiotic-treated culture was removed, mixed with 900 µL PBS, and centrifuged at 13,300 rpm to pellet surviving cells. After removing 900 µL supernatant, the pellet was resuspended in 900 µL PBS. This wash step was repeated at least twice to decrease antibiotic concentration to sub-MIC levels. Following the final wash, the supernatant was reduced to ~100 µL, and the concentrated cell suspension was transferred to a round-bottom 96-well plate. Cell suspensions were serially diluted 10-fold in PBS, and 10 µL from each dilution series was spotted onto LB agar plates to enumerate persister cells. To increase the limit of CFU detection, the entire cell suspension from the first undiluted well (~80 µL) was also plated onto LB agar. Plates were incubated at 37°C for 16 h, after which CFUs were quantified. Kill curves were generated by plotting CFU levels as a function of antibiotic treatment time. Persister fractions were calculated as the ratio of CFU at the end of treatment to the initial CFU count. All plates were retained for at least three days to ensure the detection of any late-emerging colonies. For all experiments involving overexpression plasmids, IPTG (1 mM) and kanamycin (50 µg/mL) were maintained in all cultures and agar plates.

### Cell permeability measurement

Late stationary phase cells in main cultures (*t* = 24 h) were diluted 1:100-fold in fresh LB media into 2 mL LB broth in 14-mL culture tubes. The cultures were briefly vortexed for 10 s at 200 rpm to ensure uniform cell distribution. Subsequently, 50 µL of the culture was transferred into 950 µL of sterile 0.85% NaCl solution to obtain the desired cell density for flow cytometry analysis (~10^6^ cells/mL). After adding PI (20 μM), cells were incubated in dark at 37°C for 15 min. The cells were then analyzed with a flow cytometer. Unstained live cells were used to gate the cell population on the flow cytometry diagram using forward and side scatter parameters. PI-stained dead cells obtained from ethanol (70%) treatment were used as a positive control. PI-stained live cells were used as a negative control. Cells were excited at 561-nm wavelength, and red fluorescence was detected with a 615/20-nm bandpass filter. When necessary, PI-stained cells were also analyzed with fluorescence microscopy. To image the PI-stained cells using the microscope, PBS agarose pad was generated using the method described previously (25). PI-stained cells were centrifuged and concentrated to a final volume of 100 µL and then were then spread evenly onto the PBS agarose pad and air-dried near a flame for 15 min. After drying, glass coverslips (25 × 75 × 0.17 mm) were carefully placed on top of the pads. The coverslips were secured at the corners with tape to ensure stability during imaging. Phase-contrast and red fluorescence images were captured using an EVOS FL Auto 2 fluorescence microscope (Thermo Fisher Scientific; catalog no. AMAFD2000) equipped with a 100 × oil-immersion objective (Olympus; catalog no. AMEP4733; working distance, 0.3 mm) to assess cellular morphology and membrane permeability. To further validate membrane permeability measurements, SYTOX Green (Thermo Fisher Scientific; catalog no. S7020) staining was also performed using an approach similar to the PI-based assay. Briefly, cells suspended in NaCl solution (~10^6^ cells/mL) were stained with 1 µL of SYTOX Green analyzed by flow cytometry. Cells were excited at 488 nm, and green fluorescence was detected using a 525/15-nm bandpass filter.

### Proteomic sample processing and analysis

Proteins from wild-type and *tsp* knockout strains collected at the late stationary phase (*t* = 24 h) were extracted and digested following the Sample Preparation by Easy Extraction and Digestion (SPEED) protocol (81) for subsequent LC–MS analysis. All proteomic analyses were performed at the University of Houston Mass Spectrometry Laboratory under a service agreement. Briefly, cell pellets were treated with 20 μL trifluoroacetic acid and incubated at room temperature for 5 min, followed by neutralization with 200 μL of 2 M Tris base. Tris(2-carboxyethyl) phosphine (10 mM) and 2-chloroacetamide (40 mM) were then added, and the mixture was heated at 95°C for 5 min. Proteins were digested overnight at 37°C with trypsin (1:40, wt/wt). The resulting peptides were purified using C18 ZipTips and vacuum-dried with a CentriVap concentrator (Labconco, Kansas City, MO). Dried peptides were resuspended in 2% acetonitrile (ACN) containing 0.1% formic acid (FA) for LC–MS analysis. LC–MS was performed as previously described (82) using a NanoElute LC system coupled to a timsTOF Pro mass spectrometer (Bruker Daltonics, Germany) via a CaptiveSpray source. Samples were loaded onto an in-house packed column (75 µm × 20 cm, 1.9 µm ReproSil-Pur C18 particles; Dr. Maisch GmbH, Germany) maintained at 40°C. Mobile phases consisted of buffer A (0.1% FA in water) and buffer B (0.1% FA in ACN). A short gradient was applied as follows: 0–17.8 min, 2%–30% B; 17.8–18.3 min, 30%–95% B and held at 95% B until 20.7 min. Data were acquired in parallel accumulation–serial fragmentation (PASEF) mode with four PASEF scans per cycle. The electrospray voltage was 1.4 kV, and the ion transfer tube was maintained at 180°C. Full MS scans were collected across an *m*/*z* range of 150–1,700 with a target intensity of 2.0 × 10^5^ and a minimum threshold of 2,500. The cycle time was fixed at 0.53 s, with a dynamic exclusion of 0.4 min (±0.015 amu). Only precursor ions with charge states ≥2 were selected for fragmentation. Raw MS data were analyzed using MSFragger (83) with default parameters. The UniProt-SwissProt *E. coli* K-12 database was used as reference. Cysteine carbamidomethylation was set as a fixed modification, while methionine oxidation and N-terminal acetylation were treated as variable modifications. Peptide lengths were restricted to 7–50 aa, allowing up to two missed cleavages. Both precursor and product ion masses were set to monoisotopic. The false discovery rate (FDR) was controlled below 1% at the peptide-spectrum match, peptide, and protein levels. The raw proteomics data set is provided as Supplemental data set.

### Proteomics data analysis

Processing of proteomic data and fold-change calculations followed the methods described by (48) with minor modifications. In brief, raw intensity values were organized and processed using Microsoft Excel for data transformation, normalization, fold-change computation, and statistical testing. Proteins lacking quantitative data across replicates were excluded prior to analysis. The remaining intensity values were log-transformed to approximate normal distribution. Normalization was then performed using both mean and slope correction methods to minimize technical variability within replicate groups and improve consistency between samples. Missing values were imputed using the Probabilistic Minimum Imputation approach to preserve data structure and reduce bias in downstream analysis. Following data normalization and imputation, relative protein abundance ratios between mutant and wild-type strains were computed as log₂ fold-change, and statistical significance was determined using a parametric two-tailed *t*-test. The appropriate *t*-test type (assuming equal or unequal variances) was selected based on *F*-test results assessing variance homogeneity (homoscedastic vs heteroscedastic data).

A fold-change of ≥2 was used as the criterion for upregulated proteins and ≤–2 for downregulated proteins (shown by vertical dotted lines on the *x*-axis in Fig. 4A). A *P* value ≤ 0.05, shown by a horizontal dotted line on the *y*-axis, was considered statistically significant. The upregulated genes are indicated in the green box, and green dots indicate elevated proteins. Downregulated proteins are represented by purple dots, and the purple box lists the relevant genes. The genes in each box are arranged from most upregulated to most downregulated based on the amount of fold-change. Proteins that were not statistically significant and had less than two-fold change are indicated by gray dots.

To identify significant functional associations among differentially expressed proteins, protein-protein interaction and functional enrichment analysis was performed using STRING v12.0 database (47). Differentially abundant proteins which met the selection criterion mentioned above were uploaded and mapped to the *E. coli* K-12 MG1655 proteome. STRING integrates multiple, independent evidence channels such as experimental interaction data, co-expression patterns, text-mining co-mentions, genomic neighborhood, gene fusion events, phylogenetic co-occurrence, curated pathway databases, and homology-based functional transfer to infer protein–protein associations (47). In the resulting STRING network, each line (edge) represents one or more of these evidence types. These are combined using a Bayesian framework to compute a confidence score for each functional association (47). Networks were generated using a medium—confidence interaction cutoff (score ≥ 0.4). For functional enrichment analysis, STRING performs Gene Ontology (GO) enrichment across the Biological Process, Molecular Function, and Cellular Component categories and the analyses were calculated using a hypergeometric test (47), which determines whether specific GO terms occur more frequently in the input protein set than expected based on the background *E. coli* proteome. To correct for multiple hypothesis testing, STRING applies the Benjamini–Hochberg procedure to control the false discovery rate (FDR) (47). The FDR-adjusted *P* value reflects the expected proportion of false-positive terms among all enriched categories, with lower FDR indicating higher statistical robustness (47). Resulting clusters and enrichment categories were interpreted to identify coordinated biological processes and regulatory modules. The enrichment metric used was “strength,” which is defined as the log_10_ ratio of observed to expected protein counts and higher strength corresponds to stronger overrepresentation relative to random expectation.

### Statistical analysis

Unless stated otherwise, at least three to four independent biological replicates were performed for each key assay. Each data point in the figures represents the mean ± standard deviation. Statistical analyses were performed using GraphPad Prism version 10.3.0. Statistical significance was assessed using one-way or two-way analysis of variance (ANOVA) followed by Dunnett’s post hoc test. The *P* value threshold was selected as *P < 0.05, **P < 0.01, ***P < 0.001, ****P < 0.0001.

## Data Availability

All data supporting this article are available in the main text or the supplemental material. The proteomics data set generated in this study has been deposited in the PRIDE repository under accession number PXD078436.

## SUPPLEMENTAL MATERIAL

Supplemental material spectrum.00277-26-s0001.pdf Figures S1–S14 and Tables S1-S6.

Supplemental data set spectrum.00277-26-s0002.xlsx Proteomics raw data.

ASM does not own the copyrights to Supplemental Material that may be linked to, or accessed through, an article. The authors have granted ASM a non-exclusive, world-wide license to publish the Supplemental Material files. Please contact the corresponding author directly for reuse.
