# Supplementary material for: Tail-specific protease (Tsp)-mediated envelope remodeling and beta-lactam tolerance in Escherichia coli
Source: Microbiol Spectr. 2026 Jun 26;14(8):e00277-26. doi: 10.1128/spectrum.00277-26 (PMC13435647; doi:10.1128/spectrum.00277-26)
Supplement: Supplementary file 1 [file spectrum.00277-26-s0001.pdf]

Supplementary Information

Tail-specific protease (Tsp) - mediated envelope remodeling and beta-lactam tolerance in *Escherichia coli*

Jenet Narzary[a,b], Sayed Golam Mohiuddin[a,b], Aslan Massahi[a] and Mehmet A. Orman[a,b,*]

[a]Department of Chemical and Biomolecular Engineering, University of Houston, Houston, Texas, United States.
[b]Department of Biomedical Engineering, University of Wisconsin, Madison, Wisconsin, United States.

*Corresponding author: maorman@wisc.edu

Supplementary Figures

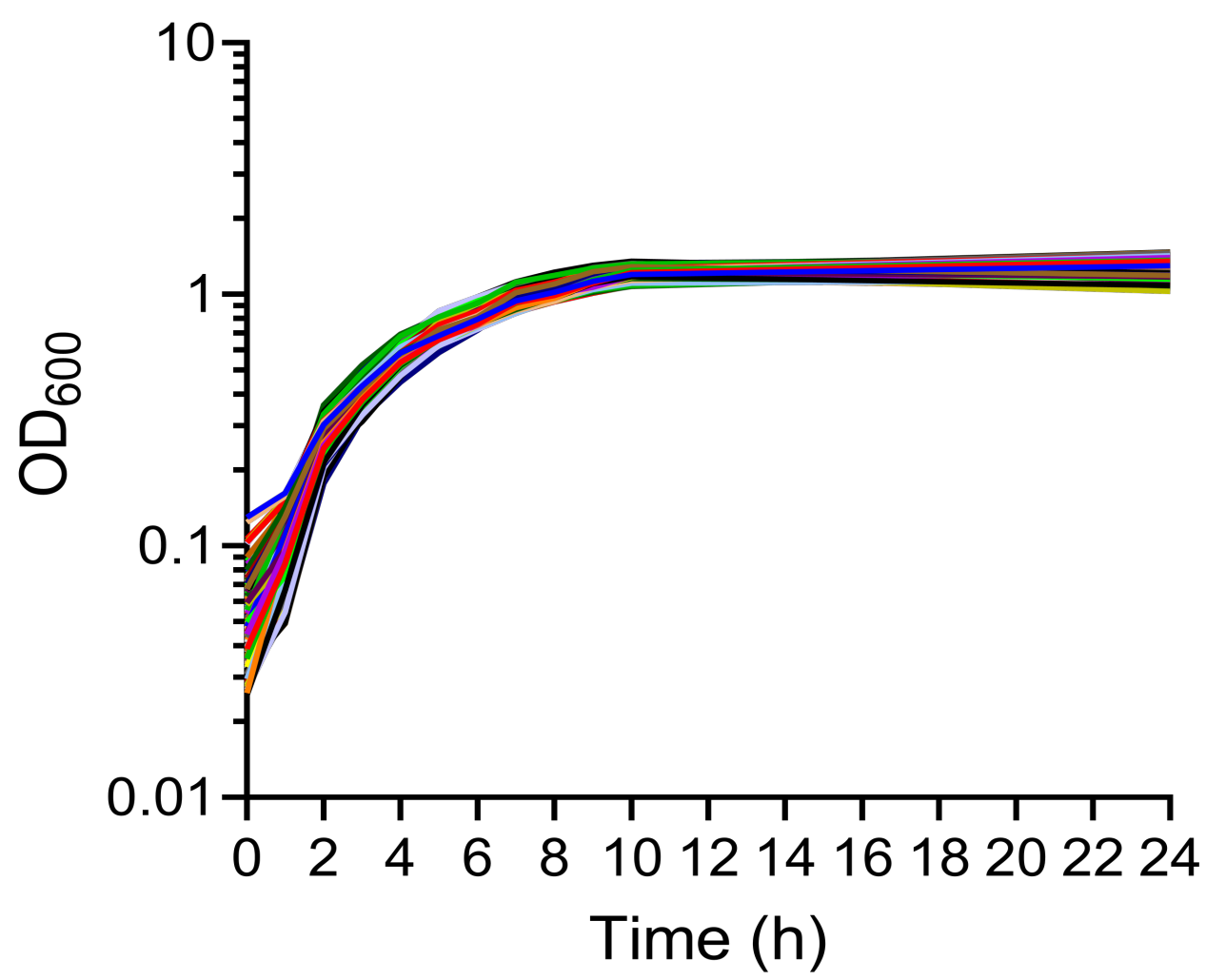

Figure S1. Growth curves of *E. coli* cultures in 96-well format. Approximately 200 promoter-GFP reporter strains, randomly selected from the library and all sharing the same MG1655 background, were grown in 96-well plates after dilution of overnight cultures (5 µL into 245 µL) into fresh medium. Optical density at 600 nm was recorded at regular intervals to monitor growth. Growth curves were highly consistent across strains, with some variability at the earliest time points, when OD_{600} values approached background levels.

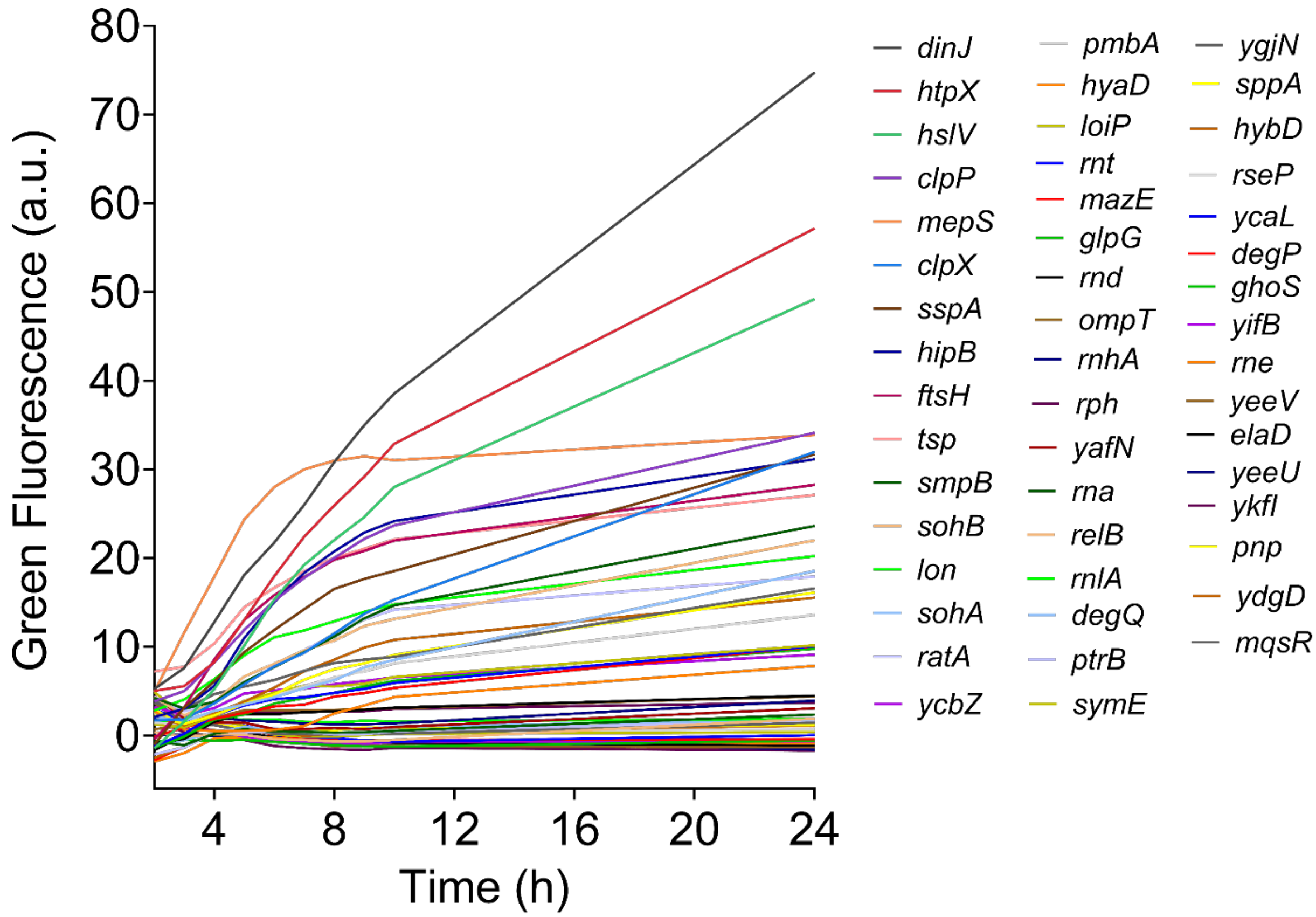

Figure S2. Time-profile plot showing the GFP expression of the promoters. The degradative enzymes from the promoter library were chosen and their GFP expression was monitored for 24 hours.

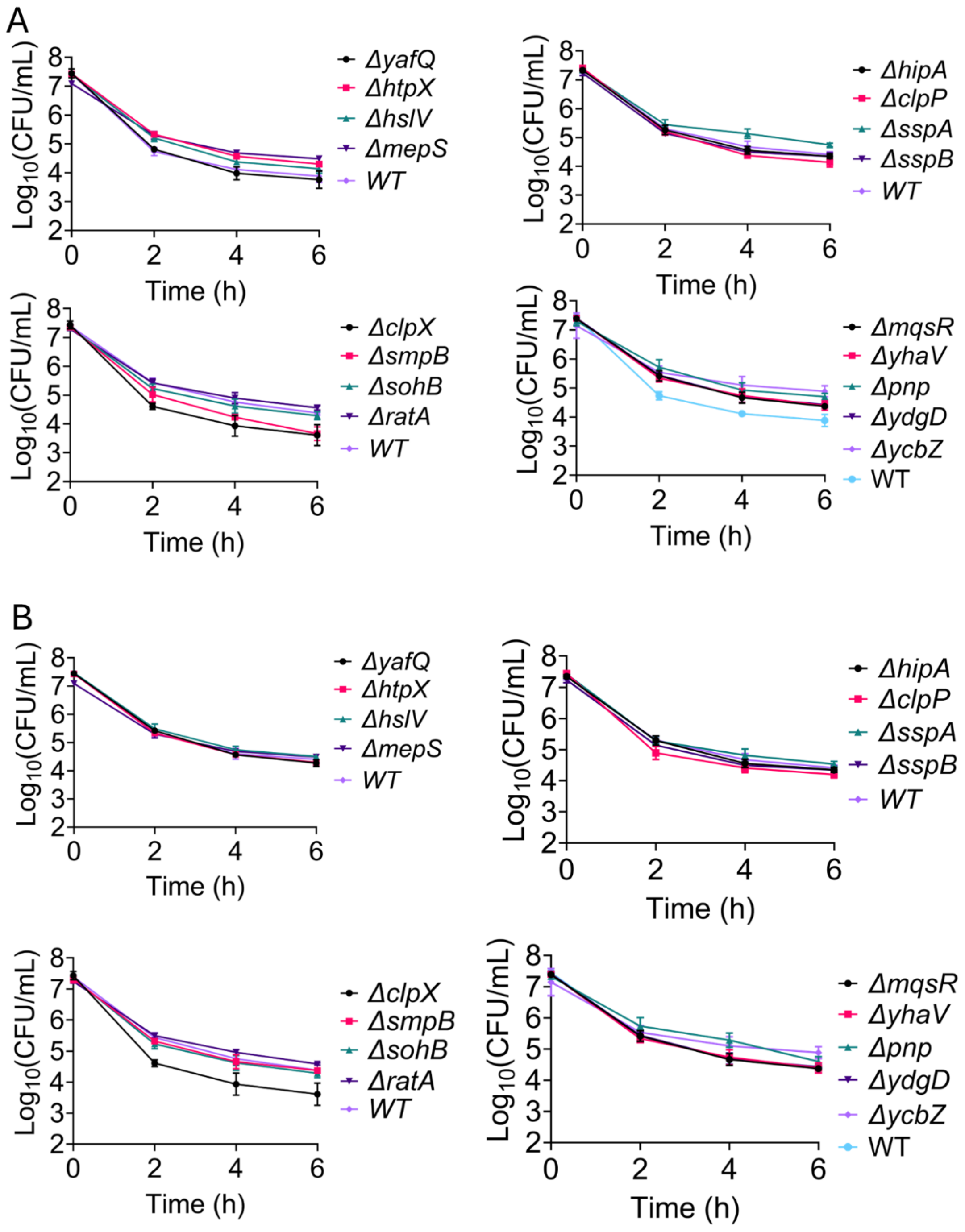

Figure S3. Kill curves of selected knockout strains. Stationary-phase cultures were diluted 100-fold into fresh LB and treated with (A) ampicillin (200 μg/mL) or (B) ofloxacin (5 μg/mL) for 6 hours. Colony-forming units (CFUs) were quantified at 0, 2, 4, and 6 hours by washing, serial dilution, and plating. Wild type (WT) was used as a control. n = 4 biological replicates; data represent mean ± standard deviation.

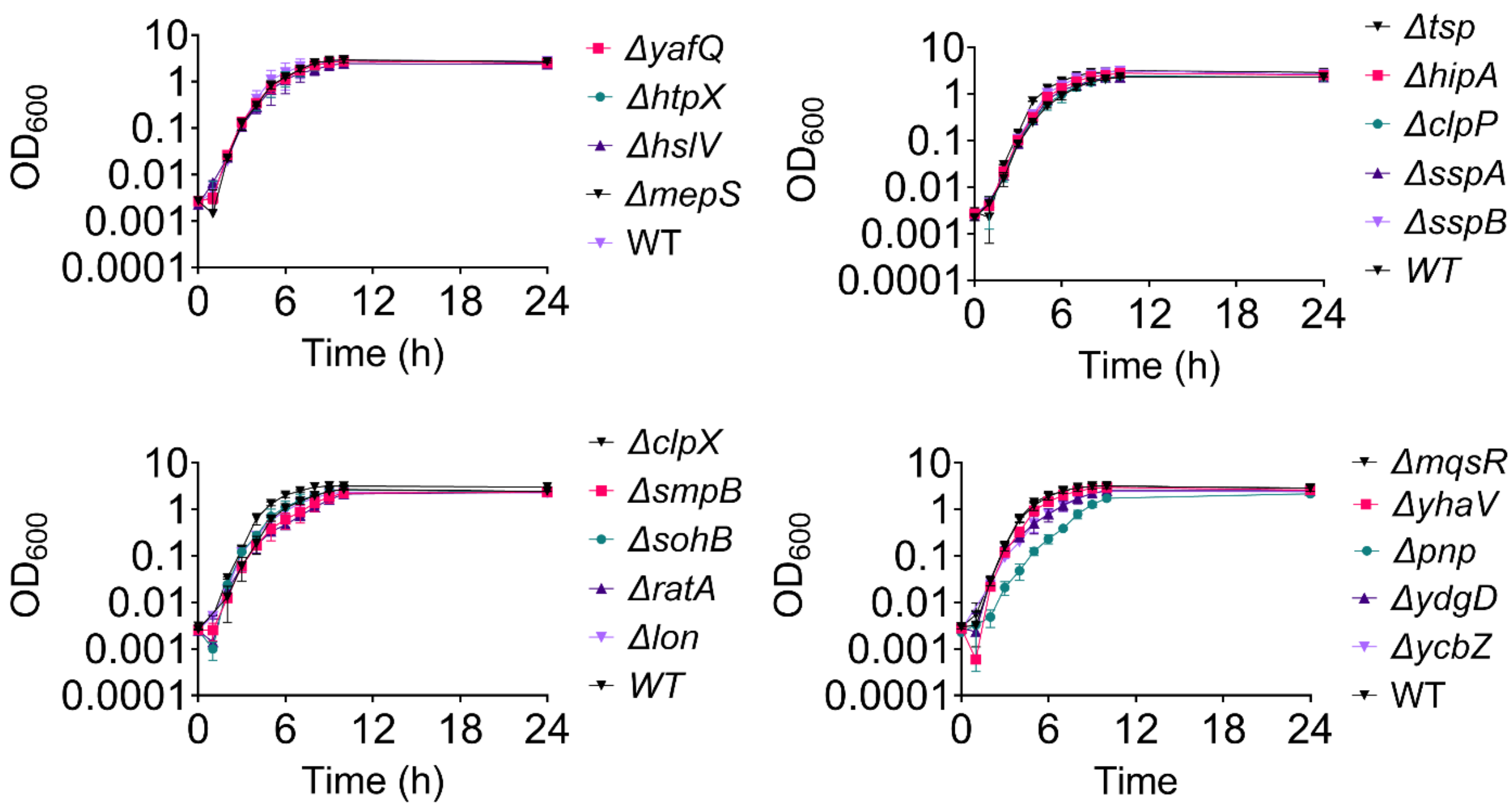

Figure S4. Growth curves of selected knockout strains. Overnight cultures of knockout strains were diluted 1,000-fold into fresh medium in falcon tubes and optical density at 600 nm was measured using a plate reader. n = 4; data are shown as mean ± standard deviation.

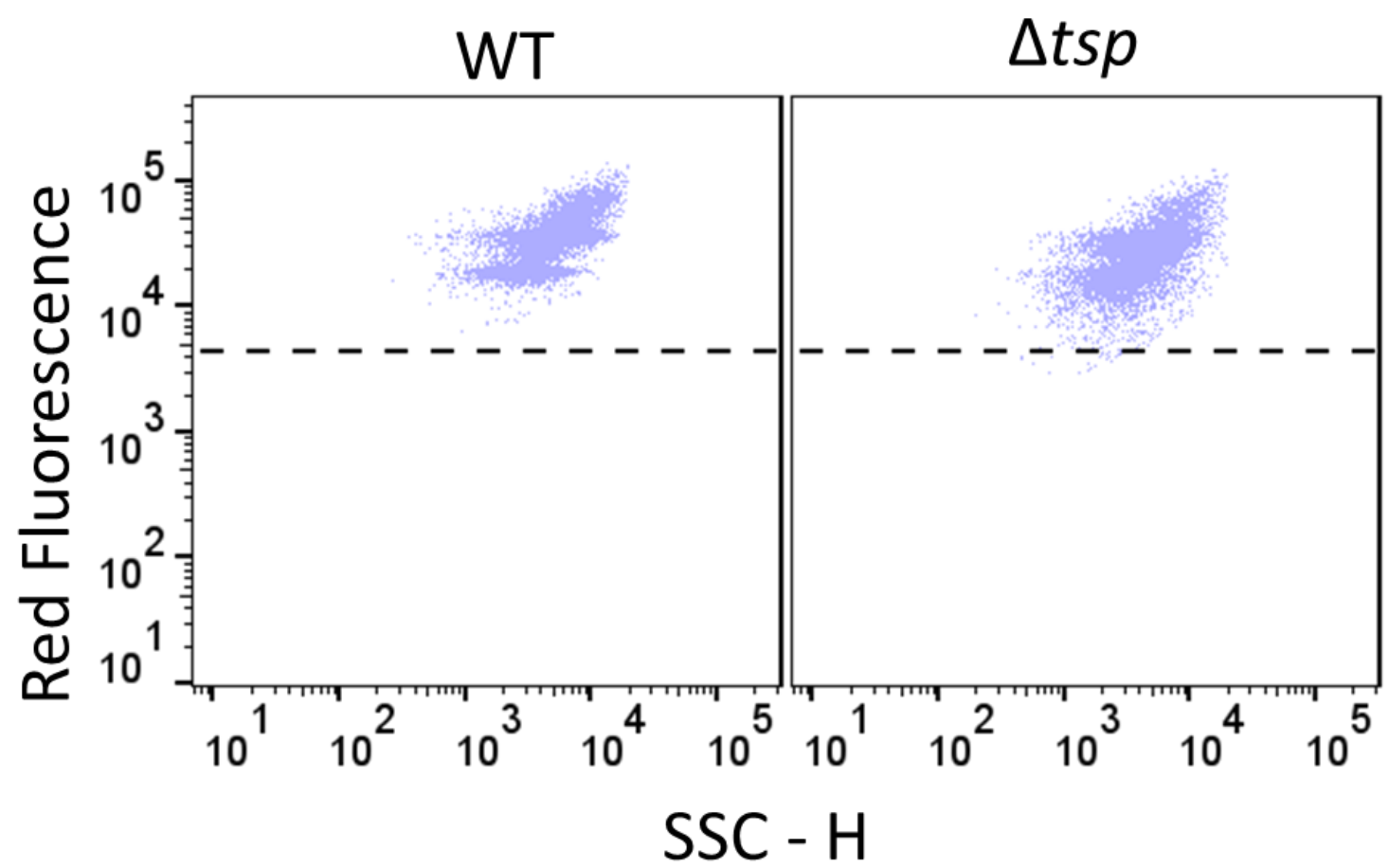

Figure S5. Dead-cell control for wild-type and Δ*tsp* strains. Late stationary-phase cultures were treated with 70% ethanol for 45 minutes, washed, stained with PI, and analyzed by flow cytometry to establish non-viable cell distributions. Side scatter height (SSC-H) represents the peak amplitude of the side-scattered light pulse generated.

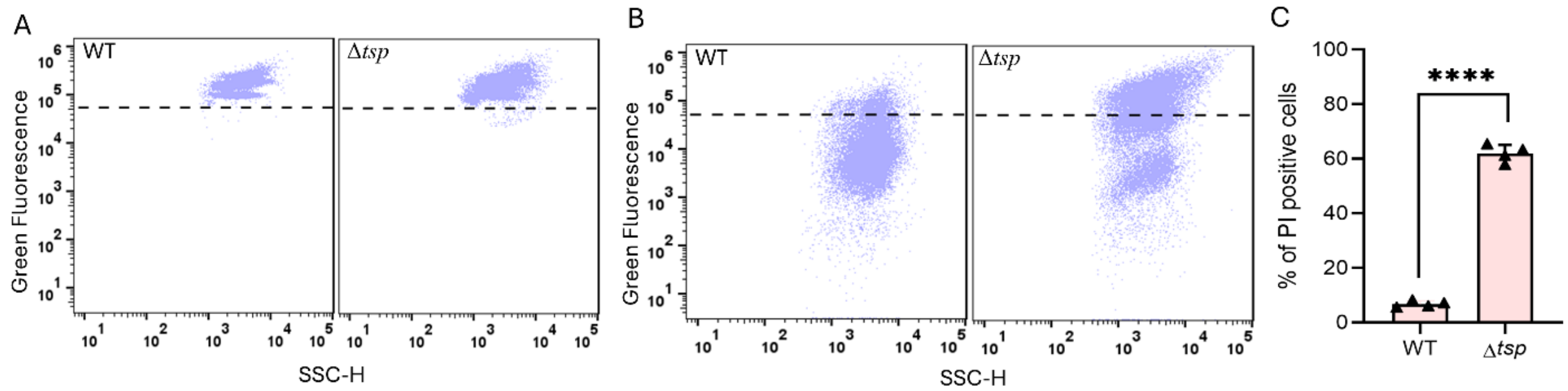

Figure S6. SYTOX Green staining assay for WT and Δ*tsp* strains. Cells suspended in NaCl solution were stained with 1 µL of SYTOX Green and analyzed by flow cytometry. Cells were excited at 488 nm, and green fluorescence was detected using a 525/15-nm bandpass filter. Side scatter height (SSC-H) represents the peak amplitude of the side-scattered light pulse generated. (A, B) Representative flow cytometry plots showing SYTOX green staining of cells analyzed at late stationary-phase (A) after treatment with 70% ethanol for 45 minutes, showing SYTOX-positive control and (B) prior to antibiotic treatment. (C) Quantification of SYTOX-positive cells at late stationary-phase prior to treatment. (****$P < 0.0001$; one-way ANOVA with Dunnett's multiple comparisons test). $n = 4$; data are shown as mean ± standard deviation.

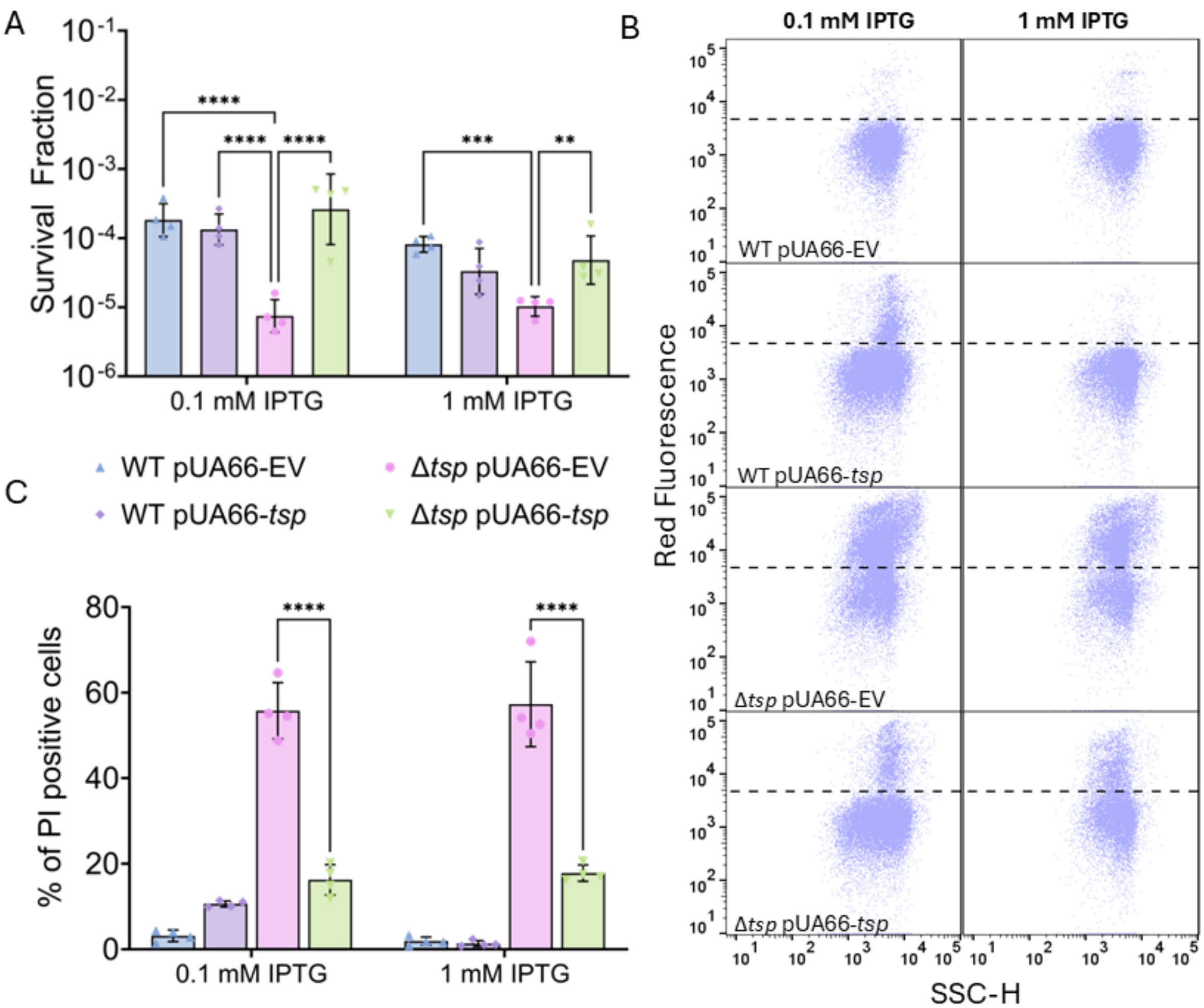

Figure S7. Complementation of Δ*tsp* restores persistence and reduces membrane permeabilization. (A) Survival fractions in persister assays. Stationary-phase cultures of wild-type cells carrying either the empty vector (pUA66-EV) or the *tsp* overexpression plasmid (pUA66-*tsp*), as well as Δ*tsp* strains carrying pUA66-EV or pUA66-*tsp*, were grown in the presence of IPTG, diluted, and treated with ampicillin for 6 h, after which survival fractions were determined. (B) Representative flow cytometry plots showing propidium iodide (PI) staining of cells analyzed at late stationary phase prior to antibiotic treatment. (C) Quantification of PI-positive cells at late stationary phase for the indicated strains. Two IPTG concentrations (0.1 or 1 mM) were tested, and IPTG was maintained throughout all experiments. Statistical significance was calculated relative to wild type (**$P < 0.01$, ***$P < 0.001$, ****$P < 0.0001$; two-way ANOVA with Dunnett's multiple comparisons test). n = 4; data are shown as mean ± standard deviation.

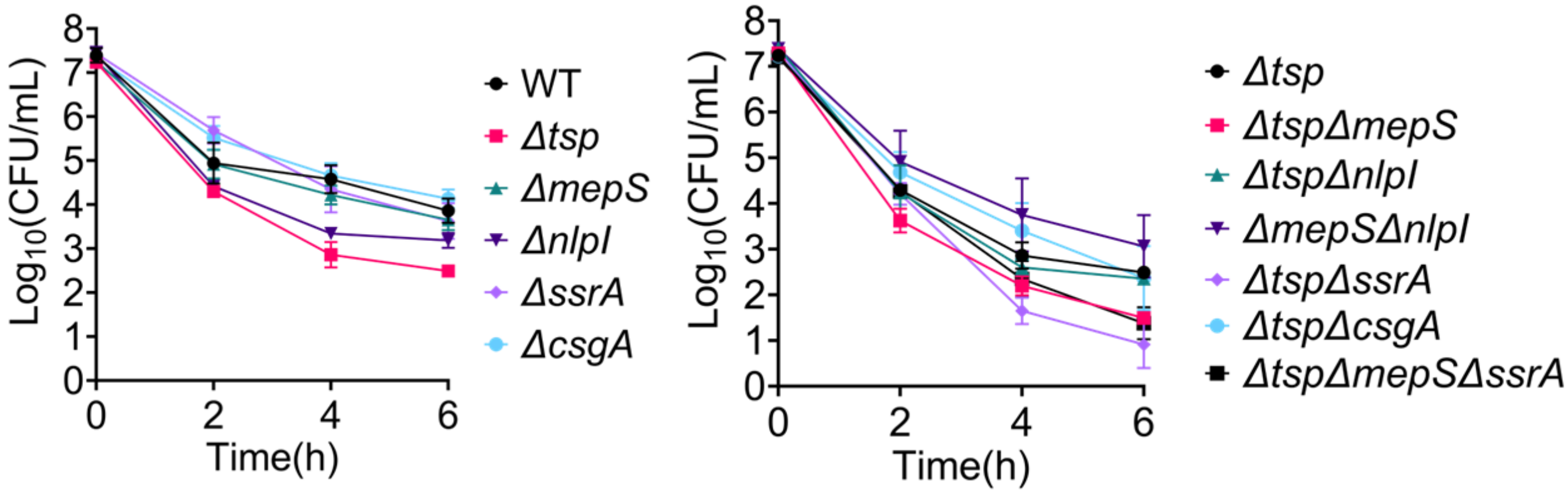

Figure S8. Kill curves for *tsp*-associated knockout strains. Stationary-phase cultures of knockout strains were diluted 100-fold into fresh medium and treated with ampicillin (200 µg/mL) for 6 hours. CFU were quantified at 0, 2, 4, and 6 hours by washing, serial dilution, and plating. n = 4; data are shown as mean ± standard deviation.

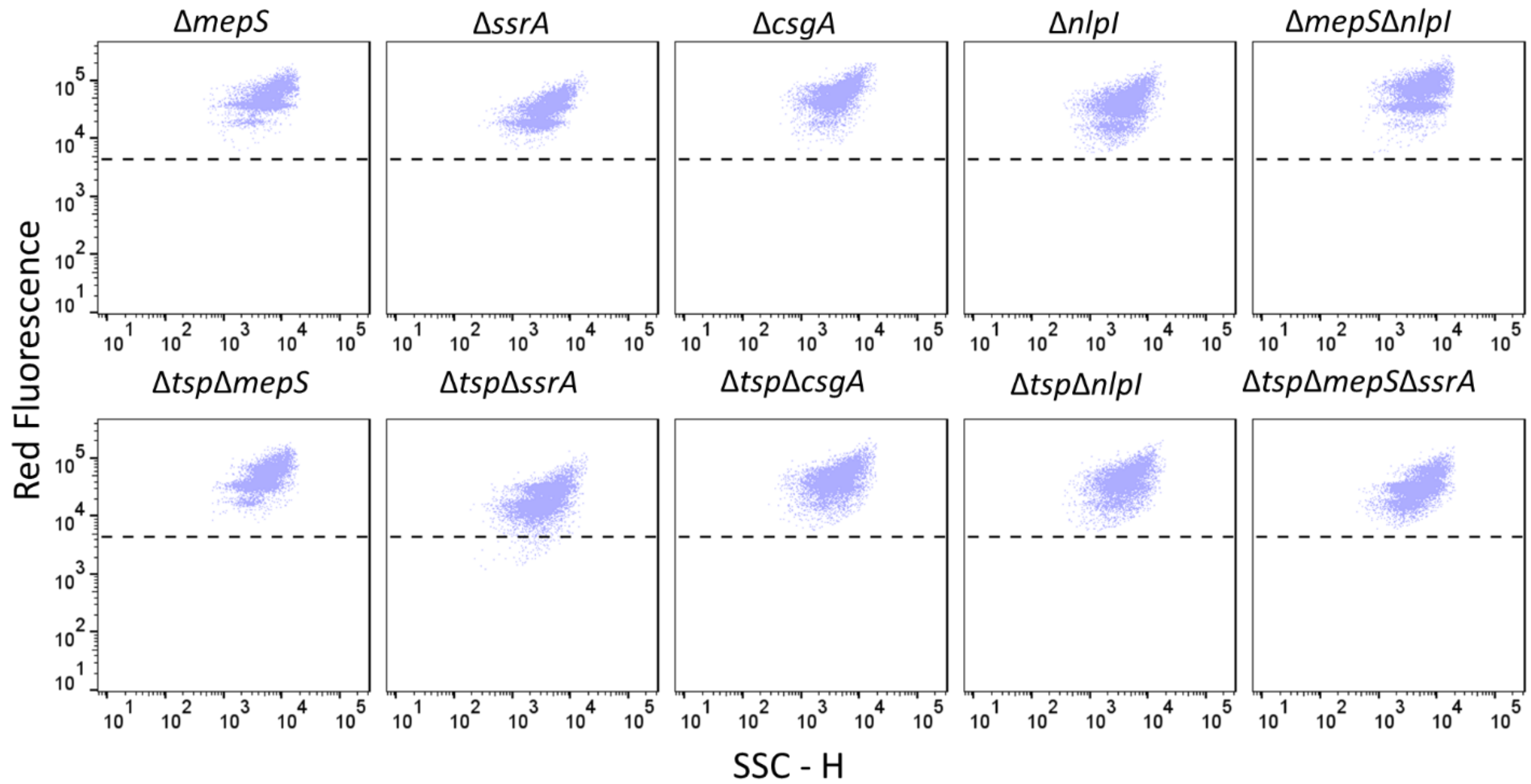

Figure S9. Dead-cell controls for *tsp*-associated knockout strains. Late stationary-phase cultures were treated with 70% ethanol, washed, stained with PI, and analyzed by flow cytometry as non-viable controls. Side scatter height (SSC-H) represents the peak amplitude of the side-scattered light pulse generated.

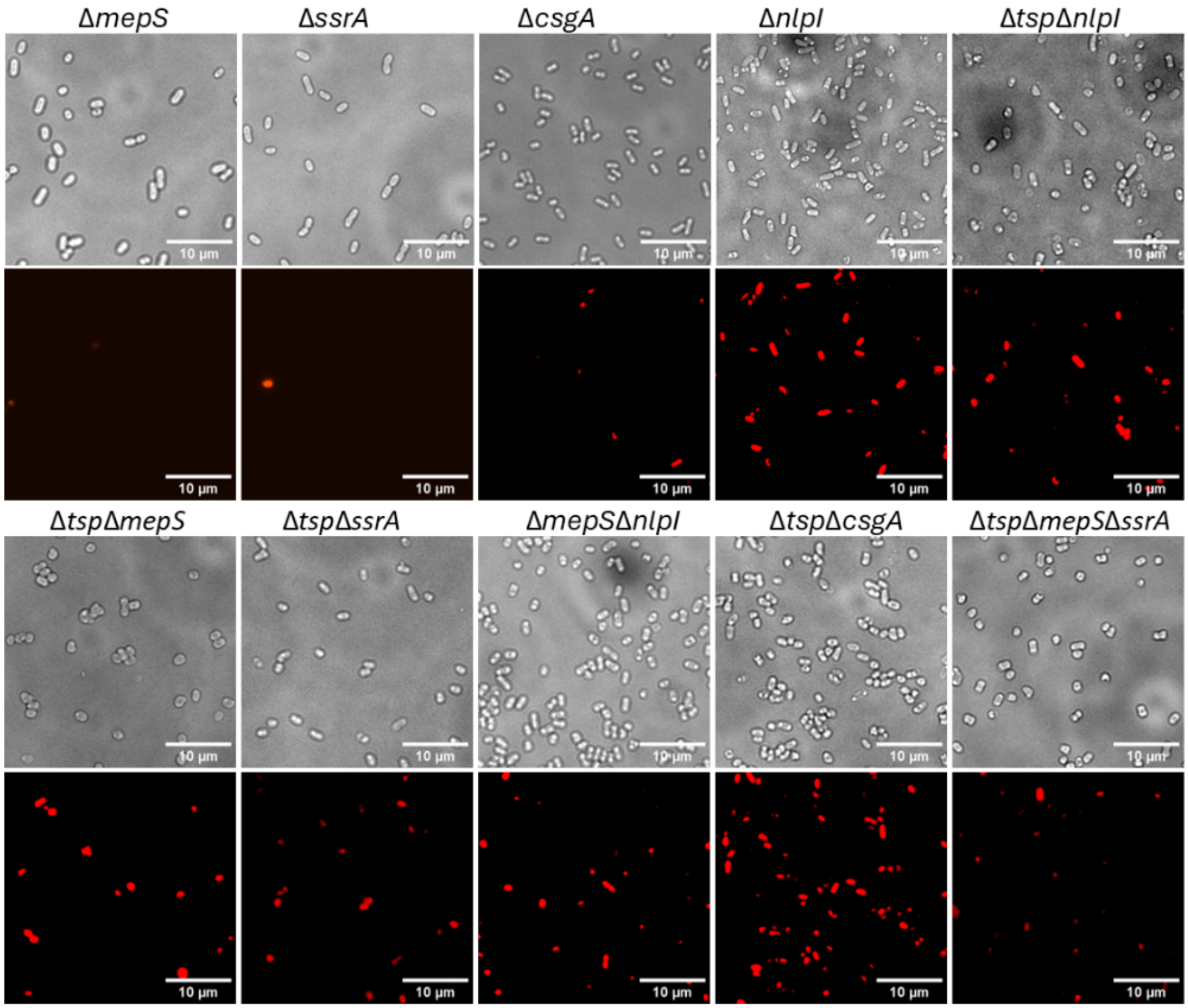

Figure S10. Microscopy analysis of envelope permeability in *tsp*-associated knockout strains. Late stationary-phase cultures were stained with PI and imaged by phase-contrast and red-fluorescence microscopy to assess envelope permeability. Representative images are shown, with consistent outcomes across replicates.

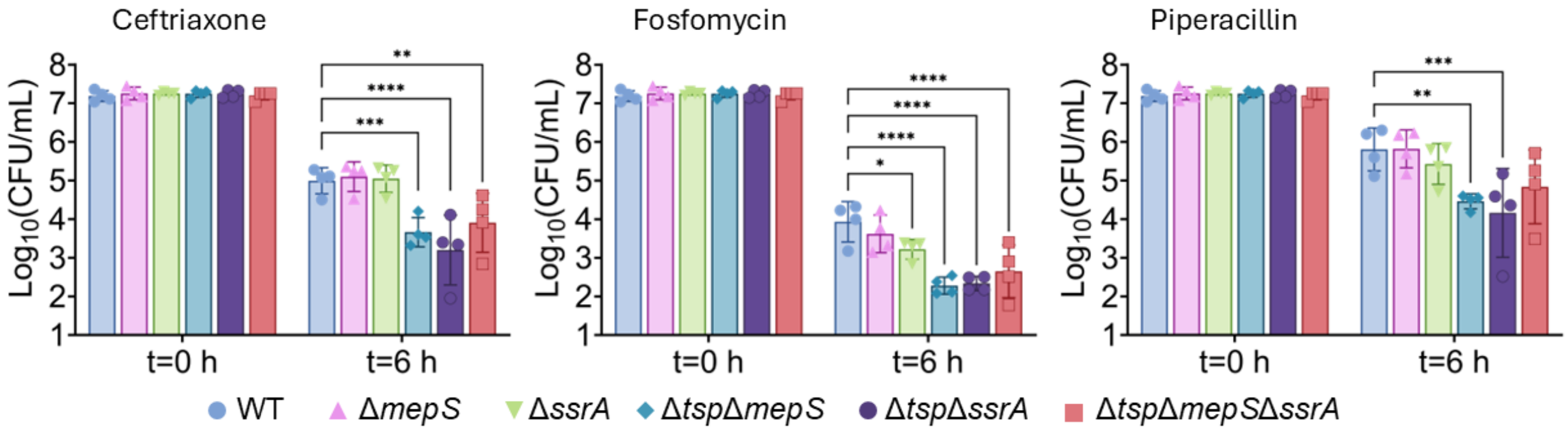

Figure S11. Survival of *tsp*-associated knockout strains exposed to cell wall–targeting antibiotics. Stationary-phase cultures were treated with ampicillin (200 µg/mL), fosfomycin (200 µg/mL), piperacillin (200 µg/mL), or ceftriaxone (5 µg/mL), and CFU were measured before and after treatment. Statistical significance was calculated relative to wild type (*$P < 0.05$, **$P < 0.01$, ***$P < 0.001$, ****$P < 0.0001$; two-way ANOVA with Dunnett's multiple comparisons test). n = 4; data are shown as mean ± standard deviation.

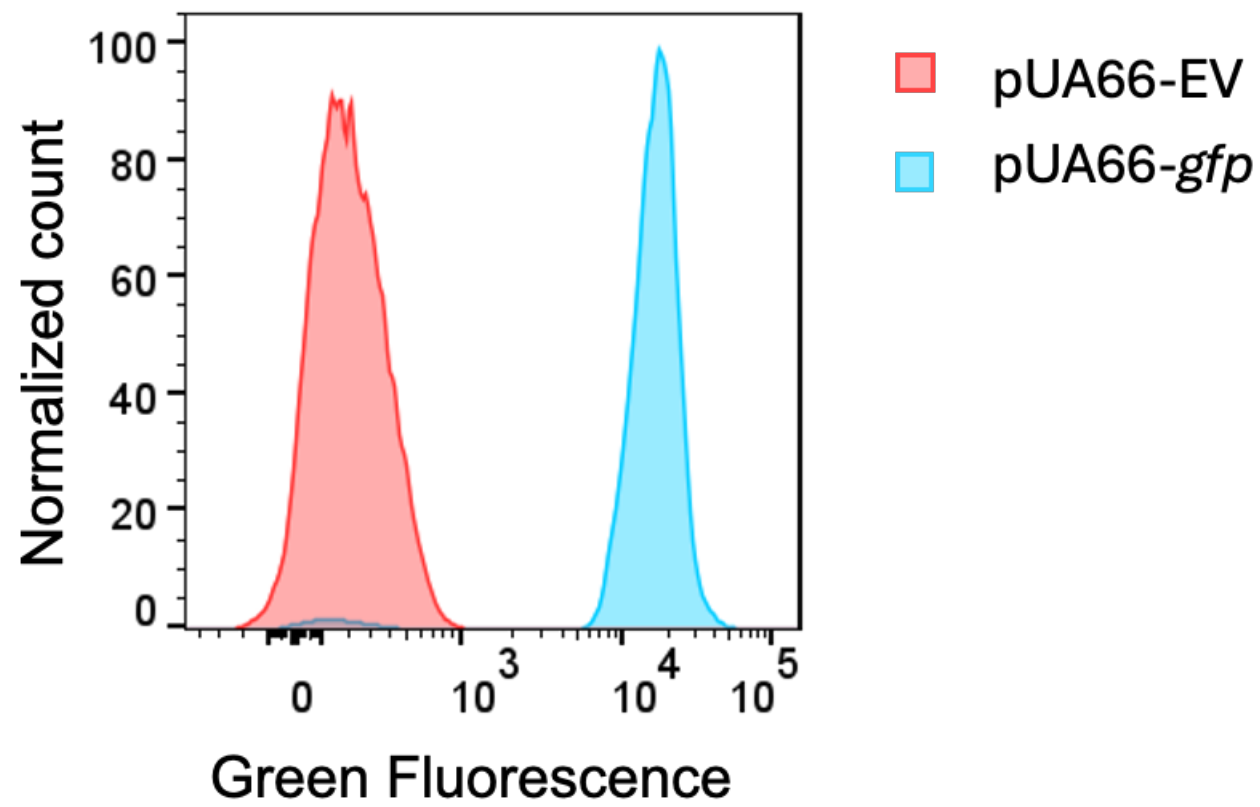

Figure S12. GFP expression from the T5 promoter. *E. coli* MG1655 strains carrying either the empty vector (pUA66-EV) or a GFP-expressing plasmid (pUA66-*gfp*) were grown in the presence of 1 mM IPTG. GFP levels were measured in late stationary phase by flow cytometry. Cells harboring pUA66-*gfp* exhibited a clear increase in GFP fluorescence relative to the empty vector control, confirming that the T5 promoter and plasmid backbone reliably drive protein expression under the induction conditions used in this study.

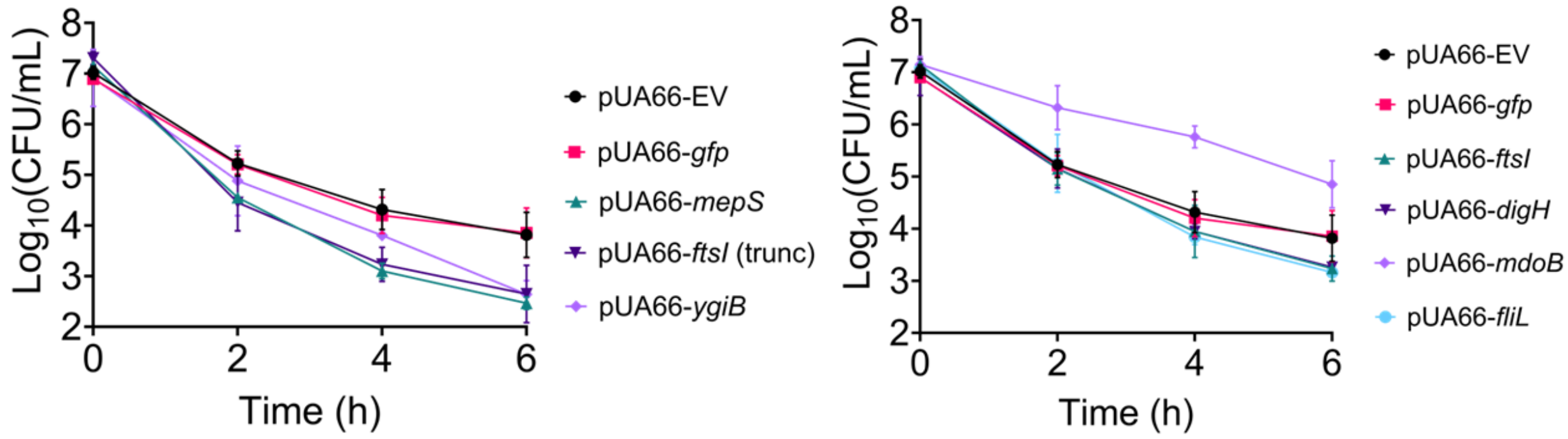

Figure S13. Kill curves for overexpression strains. Stationary-phase cultures harboring overexpression plasmids were diluted 100-fold into fresh medium and treated with ampicillin (200 µg/mL) for 6 hours. CFU were quantified at 0, 2, 4, and 6 hours. n = 4; data are shown as mean ± standard deviation.

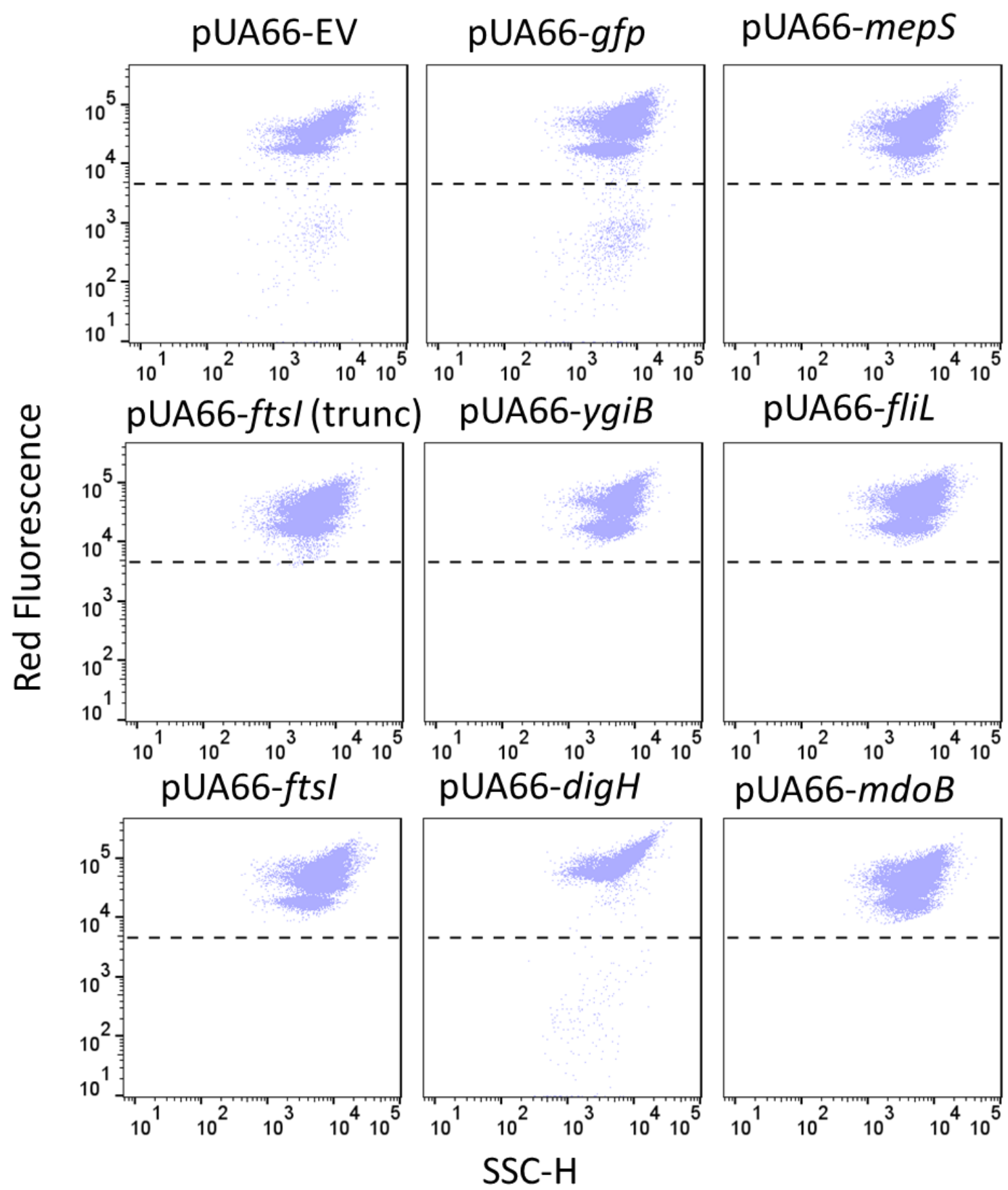

Figure S14. Dead-cell controls for overexpression strains. Late stationary-phase cultures of overexpression strains were treated with 70% ethanol, washed, stained with PI, and analyzed by flow cytometry to define non-viable reference distributions.

Supplementary Tables

Table S1: Summary of functional roles for the selected degradative enzymes

**Promoter**	**Gene name**	**Functions** [a]
P_{dinJ}	*dinJ*, ***yafQ***	An interferase toxin of the YafQ-DinJ toxin-antitoxin system that inhibits translation by interacting with the 50S subunit of the ribosome.
P_{htpX}	*htpX*	A membrane-associated heat shock protein implicated in degrading misfolded proteins and its expression is dictated by CpxR-CpxA signal system.
P_{hslV}	*hslV*	Constitutes the peptidase unit of the HslVU protease, which is part of the AAA+ protease family.
P_{clpP}	*clpP*	A chymotrypsin-like serine protease that constitutes the peptidase component of the ClpAP, ClpAPX, and ClpXP protease complexes, which is part of the AAA+ protease family.
$P_{spr\ (mepS)}$	*mepS*	A DD-endopeptidase associated with murein incorporation during cell growth.
P_{clpX}	*clpX*	An ATP-dependent chaperone for binding substrates to the ClpP catalytic core in both the ClpXP and ClpAXP protease systems
P_{sspA}	*sspA*	A transcriptional regulator associated with RNA polymerase that is induced in response to nutrient starvation.
P_{sspA}	*sspB*	A specificity-enhancing factor for ClpXP protease that binds to SsrA tag.
P_{hipB}	*hipB*, ***hipA***	A kinase associated with mediating persistence by activating ppGpp synthesis and serves as toxin component of HipAB toxin–antitoxin module.
P_{ftsH}	*ftsH*	An ATP-dependent zinc metalloprotease that forms the FtsH–HflKC membrane complex and degrades both misfolded inner-membrane proteins and selected soluble cytoplasmic substrates.
P_{proQ}	*proQ*, ***tsp***	A periplasmic protease with specificity for C-terminal processing, involved in degradation of SsrA-tagged proteins and regulation of the endopeptidase protein MepS.
P_{smpB}	*smpB*	A component of SsrA-tagging complex that is essential for trans-translation.
P_{sohB}	*sohB*	An inner membrane protein resembling the signal peptide peptidase protease IV
P_{lon}	*lon*	An intracelluar protease which is part of the AAA+ protease family and is important for protein quality control.

$P_{sohA\ (prlF)}$	*yhaV*	An mRNA endonuclease of the RelE family and serves as the toxin component of the YhaV–PrlF type II toxin–antitoxin module known to associate with 70S ribosome and the 50S ribosomal subunit.
$P_{yfjG\ (ratA)}$	*ratA*	A toxic small protein that binds the free 50S ribosomal subunit and blocks 70S ribosome assembly leading to inhibition of translation initiation.
$P_{ygiU\ (mqsR)}$	*mqsR*	A mRNA interferase that inhibits translation by sequence-specific cleavage at GCU motifs.
P_{pnp}	*pnp*	An exonuclease that exhibits 3′ terminal polymerase activity, involved in mRNA decay and RNA processing.
P_{ydgD}	*ydgD*	A predicted periplasmic serine protease that may participate in protein quality control in the cell envelope.
P_{ycbZ}	*ycbZ*	A putative ATP-dependent protease shown to genetically interact with several translation and ribosome biogenesis factors.

[a]Source: Keseler, I. M., Collado-Vides, J., Gama-Castro, S., Ingraham, J., Paley, S., Paulsen, I. T., Peralta-Gil, M., & Karp, P. D. (2005). EcoCyc: a comprehensive database resource for Escherichia coli. *Nucleic acids research*, *33*(Database issue), D334–D337. https://doi.org/10.1093/nar/gki108

Table S2: Minimum inhibitory concentrations (MICs) of various β-lactam antibiotics against wild-type and the knockout strains exhibiting reduced persistence.

**Strain**	**MIC Range (μg/mL)**			
	**Ampicillin**	**Ceftriaxone**	**Piperacillin**	**Fosfomycin**
E. coli K-12 MG1655 Wild Type (WT)	4-6	0.012-0.023	0.5-0.75	0.75-1
E. coli K-12 MG1655 Δ*tsp*	3-4	0.023-0.032	0.5-0.75	1-1.5
E. coli K-12 MG1655 Δ*tsp*Δ*mepS*	0.5-0.75	0.012-0.023	0.5-0.75	0.75-1
E. coli K-12 MG1655 Δ*tsp*Δ*ssrA*	0.75-1	0.012-0.016	0.38-0.5	0.75-1
E. coli K-12 MG1655 Δ*tsp*Δ*mepS*Δ*ssrA*	0.75-1	0.012-0.016	0.38-0.5	0.75-1

Table S3: Summary of differentially expressed proteins showing significant upregulation in the Δ*tsp* strain compared with WT.

Protein	Log_2(Fold change)	Protein description	Location	More details[a]
MepS / Spr	8.959361	Murein DD-endopeptidase	Periplasmic	Peptidoglycan endopeptidase with specificity for D-ala-m-DAP (4→3) cross links
DigH / YddW	5.844179	Divisome localized glycosyl hydrolase	Outer membrane	Localizes to outer membrane cleaves peptidoglycan to disaccharides and aggregates in absence of *prc*
FliK	4.800611	Flagellar hook-length control protein	Cytoplasm	Constitutes the soluble part of flagellar export apparatus and associated with *flhB* for substrate specificity switching
FliL	3.767962	Flagellar protein	Inner membrane	Flagellar motor component thought to support torque generation and stable rotation
YncE	3.752688	Uncharacterized protein YncE	Periplasmic	Hypothesized to be involved in iron acquisition and likely exported through the Sec pathway
MdoB	3.751981	Phosphoglycerol transferase I	Inner membrane	Transfers phosphoglycerol onto periplasmic glucans and is strongly induced under low-osmolarity conditions to promote osmoregulated glucan biosynthesis
CpxP	3.681145	Periplasmic protein involved in stress resistence	Periplasmic	Stress-response chaperone that binds misfolded proteins for delivery to DegP and during absence of misfolded proteins, it suppresses the Cpx envelope stress pathway by interacting with CpxA.
FlgA	3.661906	Flagella basal body P-ring formation protein	Periplasmic	Assists in assembling the P-ring of the flagellar basal body, a periplasmic structural component required for flagellar stability
YffR	3.58023	Uncharacterized protein	Unknown	No information

PspE	3.328801	Thiosulfate sulfurtransferase	Periplasmic	Catalytic activity for transferring sulfur from thiosulfate to a thiol compound
YgiB	3.19326	UPF0441 protein	Outer membrane	Induced upon biofilm formation and expression was found to be dependent on the presence of the F plasmid
AmiB	3.166484	N-acetylmuramoyl-L-alanine amidase	Periplasmic	Has pH-dependent activity and primary function as amidase is to split peptide stems from glycan strands and is associated with the onset of envelope constriction during cell division
Gcd	3.164725	Quinoprotein glucose dehydrogenase	Periplasmic	PQQ-dependent glucose dehydrogenase that oxidizes glucose and shuttles electrons into the respiratory chain, although without generating a proton gradient
YcfJ	3.126307	Uncharacterized protein	Inner membrane	Upregulated during biofilm growth and expression was found to be dependent on the presence of the F plasmid. Resembles UmoD from *Proteus mirabilis*, which is linked to motility, swarming, and cell elongation.
FtsQ	2.880081	Cell division protein	Inner membrane	A core divisome protein required throughout septal formation, acting as a scaffold for additional division components
YehD	2.788903	Uncharacterized fimbrial-like protein	Outer membrane	Autotransporter like protein and yeh operon promotes biofilm formation in minimal media on abiotic surfaces
YfgG	2.698508	Uncharacterized protein	Inner membrane (predicted)	Predicted inner membrane protein implicated in Ni^{2+}/Co^{2+} stress responses.
FtsB	2.660195	Cell division protein	Inner membrane	Divisome component whose recruitment depends on FtsQ and FtsL and is required to localize FtsI and FtsW to the Z-ring.

YobA	2.415026	Copper-binding protein	Periplasmic	Delivers copper to membrane-associated targets, including NDH-2
MrcA	2.36536	Penicillin-binding protein 1A (PBP 1A)	Inner membrane	Bifunctional peptidoglycan synthase with transglycosylase and transpeptidase activity, central to cell wall formation
YahO	2.184714	Uncharacterized protein	Outer membrane	σS-regulated lipoprotein whose function remains unclear but is expressed during stationary phase and stress
HslJ	2.066211	Heat shock protein	Periplasmic	Heat shock lipoprotein linked with Novobiocin resistance
YgiW	2.035864	Uncharacterized protein	Periplasmic	Involved in responses to peroxide and cadmium; requires SecB for proper targeting during export
YnjE	2.035246	Thiosulfate sulfurtransferase	Inner membrane	Likely involved in sulfur handling and linked to ABC transporter complex
OsmB	1.988496	Osmotically inducible lipoprotein B	Outer membrane	Osmotically inducible outer membrane lipoprotein expressed under high-osmolarity conditions
FlhB	1.911248	Flagellar biosynthetic protein	Inner membrane	Flagellar export apparatus required for secretion of structural flagellar components and works in conjunction with Flik
MltA	1.788164	Membrane-bound lytic murein transglycosylase A	Periplasmic	Overexpression weakens the cell wall and causes spheroplasting and lysis of cells grown at 30° C
YieE	1.784309	Uncharacterized protein	Inner membrane (predicted)	No information
HiuH / YedX	1.764241	5-hydroxyisourate hydrolase	Cytoplasm	Cleaves 5-hydroxyisourate as part of purine degradation
FtsI	1.745594	Peptidoglycan D, D-transpeptidase (PBP3)	Inner membrane	Essential for synthesizing peptidoglycan during cytokinesis
AraF	1.622514	L-arabinose-binding	Periplasmic	Binding protein for the arabinose ABC transporter

		periplasmic protein		
BcsG	1.548759	Cellulose biosynthesis protein/ Cellulose phosphoethanolamine transferase	Inner membrane	Associated with the cellulose synthase complex for adding phosphoethanolamine modifications to cellulose
YcfL	1.511434	Uncharacterized protein	Outer membrane	Part of OMP family
YjcO	1.480299	Uncharacterized protein	Inner membrane (predicted)	No information
DedD	1.433245	Cell division protein D	Inner membrane	Divisome component that interacts with PBP1A and PBP1B and stimulates their glycosyltransferase activities.
DacA	1.375822	D-alanyl-D-alanine carboxypeptidase (PBP5)	Periplasmic	D-Ala-D-Ala carboxypeptidase that cleaves the carboxy-terminal D-alanine from peptidoglycan pentapeptides and is linked to intrinsic β-lactam resistance in *E. coli* K-12.
YbiS / LdtB	1.322677	L, D-transpeptidase	Periplasmic	L, D-transpeptidase that forms 3→3 crosslinks or links mDAP residues to the C-terminal Lys of Braun lipoprotein
FecB	1.29922	Fe(3+) dicitrate-binding periplasmic protein	Periplasmic	Required for the uptake of ferric dicitrate via the Fec transport system
YhgE	1.238447	Putative transport protein	Periplasmic (predicted)	No information
YidQ	1.208655	Uncharacterized protein	Inner membrane (predicted)	Regulated by σE, suggesting involvement in extracytoplasmic or envelope stress responses.
SbmA	1.173074	Peptide antibiotic transporter	Inner membrane	Transport protein energised by the electrochemical gradient and can import certain peptide antibiotics

EmtA / MltE	1.171656	Lytic murein transglycosylase E	Inner membrane	Cleaves the β-1 → 4 glycosidic bond between N-acetylglucosamine (GlcNAc) and N-acetylmuramic acid (MurNAc), generating 1,6-anhydroMurNAc ends in peptidoglycan fragments
RseA	1.147689	Anti-sigma-E factor	Inner membrane	Anti-σE factor degraded by DegS and RseP during extracytoplasmic stress, induced by misfolded proteins.
ErfK / LdtA	1.121207	L, D-transpeptidase	Periplasmic	σS-dependent and upregulated in response to the stationary phase transition, osmotic upshift, and acid stress
YphF	1.103863	ABC transporter periplasmic-binding protein	Inner membrane (predicted)	Predicted to be substrate-binding component of an ABC transporter, though its transported substrate is unknown
DamX	1.098572	Cell division protein	Inner membrane	Non-essential cell division protein that contains a C-terminal SPOR domain which targets the protein to the septal ring
MltG / YceG	1.083383	Endolytic murein transglycosylase	Inner membrane	Enzyme with endolytic murein transglycosylase activity implicated in termination of nascent peptidoglycan (PG) synthesis
YbaV	1.072337	Helix-hairpin-helix 3 family protein	Periplasmic (predicted)	Contains motif that may bind to DNA and is identified among genes involved in repressing cell-to-cell plasmid transfer
BsmA / YjfO	1.05898	Lipoprotein BsmA	Inner membrane	Upregulated during biofilm growth on serine limited defined medium and its mutants show reduced microcolony formation and increased motility, with diminished resistance to acid and peroxide
EptC / YijP	1.045039	Phospho-ethanolamine transferase	Inner membrane	Involved in incorporation of phosphoethanolamine (P-EtN) onto the phosphorylated heptose I residue of E. coli K-12 lipopolysaccharide, modifying outer membrane structure

RcnB / YohN	1.040524	Nickel/cobalt homeostasis protein	Periplasmic	Linked to nickel/cobalt stress resistance and induced in response to three cationic amphipathic antimicrobial peptides (AMPs)
YecD	1.031312	Uncharacterized iso-chorismatase family protein	Inner membrane (predicted)	No information
FhuE	1.023037	Ferric coprogen/ferric rhodotorulic acid outer membrane transporter	Outer membrane	TonB-dependent receptor that imports ferric coprogen and ferric rhodotorulic acid into the periplasm
RplW	1.009545	50S ribosomal subunit protein L23	Cytoplasm	Essential for growth of *E. coli* and provides a docking site for chaperones to assist in co-translational folding

[a]Source: Keseler, I. M., Collado-Vides, J., Gama-Castro, S., Ingraham, J., Paley, S., Paulsen, I. T., Peralta-Gil, M., & Karp, P. D. (2005). EcoCyc: a comprehensive database resource for Escherichia coli. *Nucleic acids research*, *33*(Database issue), D334–D337. https://doi.org/10.1093/nar/gki108

Table S4: Bacterial strains and plasmids used in this study.

**Bacterial strains**	**Source**
E. coli K-12 MG1655 Wild Type (WT)	Gift from Dr. Mark P. Brynildsen
E. coli K-12 MG1655 Δ*tsp*	This study
E. coli K-12 MG1655 Δ*mepS*	This study
E. coli K-12 MG1655 Δ*nlpI*	This study
E. coli K-12 MG1655 Δ*ssrA*	This study
E. coli K-12 MG1655 Δ*csgA*	This study
E. coli K-12 MG1655 Δ*rmf*	This study
E. coli K-12 MG1655 Δ*yafQ*	This study
E. coli K-12 MG1655 Δ*htpX*	This study
E. coli K-12 MG1655 Δ*hslV*	This study
E. coli K-12 MG1655 Δ*clpP*	This study
E. coli K-12 MG1655 Δ*smpB*	This study
E. coli K-12 MG1655 Δ*sohB*	This study
E. coli K-12 MG1655 Δ*lon*	Mohiuddin et al., 2022[a]
E. coli K-12 MG1655 Δ*yhaV*	This study
E. coli K-12 MG1655 Δ*clpX*	This study
E. coli K-12 MG1655 Δ*sspA*	This study
E. coli K-12 MG1655 Δ*sspB*	This study
E. coli K-12 MG1655 Δ*hlpA*	This study
E. coli K-12 MG1655 Δ*ratA*	This study
E. coli K-12 MG1655 Δ*mqsR*	This study
E. coli K-12 MG1655 Δ*pnp*	This study
E. coli K-12 MG1655 Δ*ydgD*	This study
E. coli K-12 MG1655 Δ*ycbZ*	This study
E. coli K-12 MG1655 Δ*hipA*	This study
E. coli K-12 MG1655 Δ*mepS*Δ*nlpI*	This study
E. coli K-12 MG1655 Δ*tsp*Δ*csgA*	This study
E. coli K-12 MG1655 Δ*tsp*Δ*nlpI*	This study

E. coli K-12 MG1655 Δ*tsp*Δ*mepS*	This study
E. coli K-12 MG1655 Δ*tsp*Δ*ssrA*	This study
E. coli K-12 MG1655 Δ*tsp*Δ*mepS*Δ*ssrA*	This study
**Plasmids**	**Source**
pUA66-Empty Vector (EV)	Gift from Dr. Mark P. Brynildsen
pUA66-*gfp*	Gift from Dr. Mark P. Brynildsen
pUA66-*mepS*	This study
pUA66-*ftsI* (truncated)	This study
pUA66-*ygiB*	This study
pUA66-*fliL*	This study
pUA66-*ftsI*	This study
pUA66-*digH*	This study
pUA66-*mdoB*	This study

[a] Mohiuddin SG, Massahi A, Orman MA.2022.lon Deletion Impairs Persister Cell Resuscitation in Escherichia coli. mBio13:e02187-21.https://doi.org/10.1128/mbio.02187-21

Table S5: Oligonucleotides to generate gene deletions and plasmids construction.

**Gene deletion primers for the Datsenko - Wanner method**		
**Mutations**	**Forward Primer (5’ to 3’)**	**Reverse Primer (5’ to 3’)**
Δ*tsp*::KAN^{R}	GCAGAACACCTGGTGTTCTGAAACGGAGGCCGGGCCAGGCGTGTAGGCTGGAGCTGCTTC	TGTTAAAAAATCAGGCACAATTTCTTGTGCCTGATTGATATTAACGGCTGACATGGGAAT
Δ*mepS*::KAN^{R}	CGATATTTGTCGTTAAGGACTTCAAGGGAAAACAAACAACGTGTAGGCTGGAGCTGCTTC	ACTCGTCAGGATAGCCAAGGGATTGCATCCAAACGGTTTATTAACGGCTGACATGGGAAT
Δ*nlpI*::KAN^{R}	AACAGGACGTTCATTCAACCGTGGTCTTCGGGAGTGGGAAGTGTAGGCTGGAGCTGCTTC	ATGGCAATCAAAAAAGATTACGGGCTGATGTGTACGTCAGTTAACGGCTGACATGGGAAT
Δ*ssrA*::KAN^{R}	CATGGCGCTCATAAATCTGGTATACTTACCTTTACACATTGTGTAGGCTGGAGCTGCTTC	ACTTCATCGGATGACTCTGGTAATCACCGATGGAGAATTTTTAACGGCTGACATGGGAAT
Δ*csgA*::KAN^{R}	GCCGGGGATCCTTACGATCTGCCACCTGTCCCCTCGCC	AACAGGGCTTGCGCCCTGTTTCTGTAATACAAATGATGTATTAACGGCTGACATGGGAAT
Δ*rmf*::KAN^{R}	TTTTCTTTTCCACCAGAAACCAGTATGAGGGAAACGAGGCGTGTAGGCTGGAGCTGCTTC	AACCTCCGCAATGCGGAGGTTTCTTTTTAAAGAGACAGAATTAACGGCTGACATGGGAAT
Δ*yafQ*::KAN^{R}	GGCCAAAGACGCCGATGATTTATTTGATAAATTAGGAATTTAAATGTGTAGGCTGGAGCTGCTTC	CTGTCGGCGCAGGGTTTACAAATTTCATCGGAAGCGGGCTTTAACGGCTGACATGGGAAT
Δ*htpX*::KAN^{R}	GGGTATCGCATATTGCGTTTTGTTAAACTGAGGTAAAAAGAAAATTGTGTAGGCTGGAGCTGCTTC	GCGCGTCGATCAGGACGCGCTTTTTAGTATTTACTTCATATTAACGGCTGACATGGGAAT
Δ*hslV*::KAN^{R}	CGGGGTTTGTACTCTGTATTCGTAACCAAGGGGTCAGCTCGTGTAGGCTGGAGCTGCTTC	TGACGATTTCGCGTGGGGTCATTTCAGACATGGGAGATCCTTAACGGCTGACATGGGAAT
Δ*clpP*::KAN^{R}	CGGTACAGCAGGTTTTTTCAATTTTATCCAGGAGACGGAAGTGTAGGCTGGAGCTGCTTC	CGCCCTGGATAAGTATAGCGGCACAGTTGCGCCTCTGGCATTAACGGCTGACATGGGAAT
Δ*smpB*::KAN^{R}	GGTGTTTTCGATTTCAGATTACCGATGATTCACGACGCTTGTGTAGGCTGGAGCTGCTTC	GAGGCGCGGTGAGGAACTGGTCAATAATTGGAGTGCAGGTTTAACGGCTGACATGGGAAT
Δ*sohB*::KAN^{R}	AACTGTGAGCCAAAGCGTTGTTTAACCAAGGTGGGGACTCGTGTAGGCTGGAGCTGCTTC	ATTAAAGCAAAACGCGAGGTCTTAGCCTCGCGTTTGTCTTTTAACGGCTGACATGGGAAT
Δ*lon*::KAN^{R}	ATCTGATTACCTGGCGGAAATTAAACTAAGAGAGAGCTCTGTGTAGGCTGGAGCTGCTTC	TGCCAGCCCTGTTTTTATTAGTGCATTTTGCGCGAGGTCATTAACGGCTGACATGGGAAT

Δ*yhaV*::KAN^R	TGGACGTCAACATTGATGATGAGATTGGCGACGAATAGTGTAGGCTGGAGCTGCTTC	TGGTCAACACGAATGCGTTCGGCGGGCATTGTGAACACCATTAACGGCTGACATGGGAAT
Δ*clpX*::KAN^R	ATTTGCGTCGTCGTGTGCGGCACAAAGAACAAAGAAGAGGTTTTGACCCGTGTAGGCTGGAGCTGCTTC	GGGGAGATAAAATCCCCCCTTTTTGGTTAACTAATTGTATGGGAATGGTTAATTAACGGCTGACATGGGAAT
Δ*sspA*::KAN^R	GGCTGGTAGCAGTAAAAATTCTGACTATACCTGGAGGTTTTCGTGTAGGCTGGAGCTGCTTC	GATAGGGACGACGTGGTGTTAGCTGTGACAAATCCATACAGATTAACGGCTGACATGGGAAT
Δ*sspB*::KAN^R	AAGCAGAACGTGAAATGCGTCTGGGCCGGAGTTAATCTGTGTGTAGGCTGGAGCTGCTTC	CATTAAAAAGACAAAACAGGCCGCCTGGGCCTGTTTTGTATTAACGGCTGACATGGGAAT
Δ*ratA*::KAN^R	CCCGATGTTACCCAGCGCCGGGATAGCGTTTTTTTTACAGCAGGATAAGTGTAGGCTGGAGCTGCTTC	TGCAGGTACTGCTTCTCAGGTAGCGCATAAGCCACCTCAACGGCAATTTTAACGGCTGACATGGGAAT
Δ*mqsR*::KAN^R	CCTTTTAGGTTATAACTAAAGTAACAGGGAGGCGGGGGTTGTGTAGGCTGGAGCTGCTTC	AGAAACCATTTCTCCCTGGTGGCAAACCGGACATTTCATATTAACGGCTGACATGGGAAT
Δ*pnp*::KAN^R	TGCCGTAAGGTACTGTCTAAGAAAGAGAAAGGATATTACAGTGTAGGCTGGAGCTGCTTC	GTTAAAAGCCCCCCGCCGCAGCGGAGGGCAAATGGCAACCTTAACGGCTGACATGGGAAT
Δ*ydgD*::KAN^R	GAGGGTTTGCTTTTAATAATCATAATTACCCACCAGAGTGTGATGTGTAGGCTGGAGCTGCTTC	GATGAAGACGCTGCCCGCGCTGTCGGGCAGCGTTTGAACATTAACGGCTGACATGGGAAT
Δ*ycbZ*::KAN^R	CGGTATAATCGCGGCTTTGACAACAGACTAAAAAACATCAACTGTGTAGGCTGGAGCTGCTTC	CACGCAGGATAGCTAACACGTGTACGCTGAACAAGTCCGATTAACGGCTGACATGGGAAT
Δ*hipA*::KAN^R	GCGACGCGAAAAATGCCTCGCCAGAATCAACAGAACAGCAAAATCTGGAGTGGTAGTGTAGGCTGGAGCTGCTTC	CCAGTCGTTGGCGGTCATGATTGTCATGCTCATTAACAATGACCAAACCCCATATCTTAACGGCTGACATGGGAAT

Check primers for gene deletions

**Mutations**	**External Forward Primer**	**External Reverse Primer**	**Internal Forward Primer**	**Internal Reverse Primer**
Δ*tsp*::KAN^R	CAAGCCCTGAAGGTGAAAGC	GCTCTTCGTCCAGATCATCCT	CGAGTTAGGCGATGAACTGC	TCCGTCGGTATCGCTATCTTC
Δ*mepS*::KAN^R	TGCCTCGGCGGTAGATTATCC	GCTCTTCGTCCAGATCATCCT	GTAGCGGTTCTGCTTTCTGCAT	CTGCTGGTGGAAGCATGGACA

Δ*nlpI*::KAN^R	GCCGTATCCGTCTGAGCATT	GCTCTTCGTCCAGATCATCCT	GCACTTACGCTTGCAGGATG	GCCCAGGTAGAACTCGACAA
Δ*ssrA*::KAN^R	GGCTACATGGGTGCTAAATC	GCTCTTCGTCCAGATCATCCT	CGCTCTTAGGACGGGGATCA	GACGGACACGCCACTAACAA
Δ*csgA*::KAN^R	GCAGAGACAGTCGCAAATGG	GCTCTTCGTCCAGATCATCCT	GTAGCAGCAATTGCAGCAATCG	GGTCGCGTTGTTACCAAA
Δ*rmf*::KAN^R	GACAGTGGCGTGAATTTTGCG	GCTCTTCGTCCAGATCATCCT	AGACAAAAACGAGATCGCCTGG	GGCCATTACTACCCTGTCCG
Δ*yafQ*::KAN^R	CCTGGTTCGCATAACCCTCAC	GCTCTTCGTCCAGATCATCCT	CTTGCACAAAAGCGTCATAAGGA	CGCGTGAGTTCCAGTTCTCTC
Δ*htpX*::KAN^R	CTGGTGACTTACGCACTATCCAG	GCTCTTCGTCCAGATCATCCT	AACCTGGCCGTAATGGTCGT	TAGATCAGCGGGTTGCCGTT
Δ*hslV*::KAN^R	AGACAGCACCCTCAATCGGT	GCTCTTCGTCCAGATCATCCT	TAACGGCCATGTGGTCATCG	GATGCAAATGTCGCCTGCAA
Δ*clpP*::KAN^R	AAGCCTCTTTCGGTGTTAGC	GCTCTTCGTCCAGATCATCCT	TGACTGGCCAGGTTGAAGAC	TCAGAATCGAATCGACCAGAC
Δ*smpB*::KAN^R	CCGGCTAATCTGAGGCATAACA	GCTCTTCGTCCAGATCATCCT	AACCTGGTTCAGCGACCATC	GTGGGCGTTTTTCATGATACG
Δ*sohB*::KAN^R	TCAATCAGCCTGGGGCGATA	GCTCTTCGTCCAGATCATCCT	GGGTCAACAATCTCAGCGAAC	CAATGGCTTTTGACCCCGCT
Δ*lon*::KAN^R	TGAATGTGGGGGAAACATCC	GCTCTTCGTCCAGATCATCCT	TGATGGCAATGATGGAATCG	TGGAGATTTCACGCTCCAGAC
Δ*yhaV*::KAN^R	TGCCGACTGGGAGATGAACA	GCTCTTCGTCCAGATCATCCT	CGCTTTAGTTGCCGAAGTCGAG	TTTCTCGGGTGAGGGTTTCCC
Δ*clpX*::KAN^R	AATTGATGCCAGAGGCGCAAC	GCTCTTCGTCCAGATCATCCT	ATTCGCAACCACCTGGACGA	CGCTTCCGGCTTGCCATAAA
Δ*sspA*::KAN^R	CGGCATCGACTCACCACAATG	GCTCTTCGTCCAGATCATCCT	AGCCATCAGGTCCGCATTGT	TTTCAGCTCTTTCGCACCCG
Δ*sspB*::KAN^R	CTATCTTGCTCCGCTGCTGT	GCTCTTCGTCCAGATCATCCT	ACACCACGTCGTCCCTATCTG	GTGATCTGGCTTGTCGCCATC

Δ*ratA*::KAN^{R}	GCTTGTTAAGCGCGATGGT	GCTCTTCGTCCAGATCATCCT	CTACAGCGCGGAGCAAATGT	ACCTCTTTCGCACGAACCGTA
Δ*mqsR*::KAN^{R}	CGCTACTAATGCGCCACTTTCT	GCTCTTCGTCCAGATCATCCT	CGCACACCACATACACGTTTGA	TGGGTCTGTAAACATCCTGCCA
Δ*pnp*::KAN^{R}	CGCGGCTAATGAGAGGCTTT	GCTCTTCGTCCAGATCATCCT	TGCCCGCGTAGGTTACATCA	GTTCAGCGCAACCTGCATGA
Δ*ydgD*::KAN^{R}	TGGTGGGCCGTTATCGCTTT	GCTCTTCGTCCAGATCATCCT	TGACACGACCCAATCACCGT	AACATCAGAGGCGAACCGCT
Δ*ycbZ*::KAN^{R}	AGCGATCTCAATGTTACCGTGT	GCTCTTCGTCCAGATCATCCT	CTCTTTTATGCTGGCGAAGGC	GCTTTGCGTTCGATGTCGGT
Δ*hipA*::KAN^{R}	CCCTGACAATACCACGCTCAC	GCTCTTCGTCCAGATCATCCT	AGCCGTTATGCCAGACCGTT	CGTTGCGCCAATCAACCACT

Primers for cloning

**Plasmid construct**	**Forward Primer (5' to 3')**	**Reverse Primer (5' to 3')**
pUA66-*tsp*	GCGCATCAATTGATTAAAGAGGAGACCTCTAGTatgAACATGTTTTTTAGGCTTACCG	GCGCCTGAGCTCAGTttaCTTGACGGGAGCGGGTTGTTCC
pUA66-*mepS*	GCGCCGAATTCATTAAAGAGGAGACCTCTAGTATGGTCAAATCTCAACCGAT	GCGCCGGATCCTTAGCTGCGGCTGAGAACCC
pUA66-*ftsI* (truncated)	GCGCCGAATTCATTAAAGAGGAGACCTCTAGTATGAAAGCAGCGGCGAAAACGC	GCGCCGGATCCTTACACAAATTCATTTTTATCGCCCGTTGTCAGC
pUA66-*ygiB*	GCGCCGAATTCATTAAAGAGGAGACCTCTAGTATGAAACGGACAAAATCCATACGCCACGCATC	GCGCCGGATCCTCAGCCACCCATTGAACGAGAAGAGGTACCGG
pUA66-*fliL*	GCGCCGAATTCATTAAAGAGGAGACCTCTAGTATGACTGATTACGCGATAAGCAAGAAAA	GCGCCAAGCTTTTATCGCAGAATAAAAGCGGTATACAGCAC
pUA66-*ftsI*	GCCGGGAATTCATTAAAGAGGAGACCTCTAGTATGAAAGCAGCGGCGAAAACGCAGAAACCAAAACGTCAGG	GCCGGGGATCCTTACGATCTGCCACCTGTCCCCTCGCC
pUA66-*digH*	GCCGAATTCATTAAAGAGGAGACCTCTAGTATGGATATCTGCTCCCGAAACAAGAAATTA	GCCGGATCCTTAACTGCCCCAGCGACTTTGCAGATAGCT

pUA66-*mdoB*	GCGCCGAATTCATTAAAGAGG AGACCTCTAGTTTGTCAGAACT ACTCTCTTTCGCCCTTTTT	GCGCCAAGCTTTTACCCTTCAC GTTCTACCACTTTAATTTCCAC C

Table S6: Step-by-step procedure for plasmid construction.

Plasmid	Description
pUA66-Empty Vector (EV)	A DNA fragment containing the T5 promoter, KanR marker, pUA66 origin of replication, and *lacI*^{q} was amplified from the pUA66-*gfp* plasmid using primers engineered with BspHI restriction sites. The PCR product was subsequently digested with BspHI and self-ligated to generate the modified pUA66-*EV* plasmid lacking the *gfp* gene.
pUA66-*tsp*	The *tsp* gene was amplified from the genomic DNA of *E. coli*, using forward and reverse primers with MfeI and SacI restriction enzyme cut sites, respectively. The pUA66-*gfp* plasmid was double digested with MfeI and SacI to remove the *gfp* gene. Then, the digested *tsp* gene and plasmid were ligated to generate pUA66-*tsp*.
pUA66-*gfp*	The pUA66-*gfp* plasmid was originally constructed in the Brynildsen Lab. A *T5-gfp-lacI*^{q} DNA fragment was amplified from the pQE-80L-*gfp* vector and subsequently cloned into the pUA66 backbone. Both the amplified fragment and the pUA66 plasmid were digested with AatII and SbfI, followed by ligation to generate the final construct.
pUA66-*mepS*	The *mepS* gene was amplified from the genomic DNA of *E. coli*, using forward and reverse primers with EcoRI and BamHI restriction enzyme cut sites, respectively. The pUA66-*gfp* plasmid was double digested with EcoRI and BamHI to remove the *gfp* gene. Then, the digested *mepS* gene and plasmid were ligated to generate pUA66-*mepS*.
pUA66-*ftsI (truncated)*	The *ftsI* (truncated) gene was amplified from the genomic DNA of *E. coli*, using forward and reverse primers with EcoRI and BamHI restriction enzyme cut sites, respectively. The pUA66-*gfp* plasmid was double digested with EcoRI and BamHI to remove the *gfp* gene. Then, the digested *ftsI* (truncated) gene and plasmid were ligated to generate pUA66-*ftsI* (truncated).
pUA66-*ygiB*	The *ygiB* gene was amplified from the genomic DNA of *E. coli*, using forward and reverse primers with EcoRI and BamHI restriction enzyme cut sites, respectively. The pUA66-*gfp* plasmid was double digested with EcoRI and BamHI to remove the *gfp* gene. Then, the digested *ygiB* gene and plasmid were ligated to generate pUA66-*ygiB*.
pUA66-*fliL*	The *fliL* gene was amplified from the genomic DNA of *E. coli*, using forward and reverse primers with EcoRI and HindIII restriction enzyme cut sites, respectively. The pUA66-*gfp* plasmid was double digested with

	EcoRI and HindIII to remove the *gfp* gene. Then, the digested *fliL* gene and plasmid were ligated to generate pUA66-*fliL*.
pUA66-*ftsI*	The *ftsI* gene was amplified from the genomic DNA of *E. coli*, using forward and reverse primers with EcoRI and BamHI restriction enzyme cut sites, respectively. The pUA66-*gfp* plasmid was double digested with EcoRI and BamHI to remove the *gfp* gene. Then, the digested *ftsI* gene and plasmid were ligated to generate pUA66-*ftsI*.
pUA66-*digH*	The *digH* gene was amplified from the genomic DNA of *E. coli*, using forward and reverse primers with EcoRI and BamHI restriction enzyme cut sites, respectively. The pUA66-*gfp* plasmid was double digested with EcoRI and BamHI to remove the *gfp* gene. Then, the digested *digH* gene and plasmid were ligated to generate pUA66- *digH*.
pUA66-*mdoB*	The *mdoB* gene was amplified from the genomic DNA of *E. coli*, using forward and reverse primers with EcoRI and HindIII restriction enzyme cut sites, respectively. The pUA66-*gfp* plasmid was double digested with EcoRI and HindIII to remove the *gfp* gene. Then, the digested *mdoB* gene and plasmid were ligated to generate pUA66-*mdoB*.
